# New Ophiobolins from the Deep-Sea Derived Fungus *Aspergillus* sp. WHU0154 and Their Anti-Inflammatory Effects

**DOI:** 10.3390/md18110575

**Published:** 2020-11-20

**Authors:** Wenjuan Ding, Chokkalingam Uvarani, Fangfang Wang, Yaxin Xue, Ning Wu, Liming He, Danmei Tian, Mei Chen, Youwei Zhang, Kui Hong, Jinshan Tang

**Affiliations:** 1 International Cooperative Laboratory of Traditional Chinese Medicine Modernization and Innovative Drug Development of Chinese Ministry of Education (MOE), Institute of Traditional Chinese Medicine and Natural Products, College of Pharmacy, Jinan University, Guangzhou 510632, China; dingwenjuan@stu2018.jnu.edu.cn (W.D.); uvaranichok@jnu.edu.cn (C.U.); wangfangfang@stu2019.jnu.edu.cn (F.W.); danmeitian@jnu.edu.cn (D.T.); chenm@stu2018.jnu.edu.cn (M.C.); 2 Key Laboratory of Combinatorial Biosynthesis and Drug Discovery, Ministry of Education, School of Pharmaceutical Sciences, Wuhan University, Wuhan 430071, China; xueyaxin1996@whu.edu.cn (Y.X.); wuning1999@whu.edu.cn (N.W.); heliming@whu.edu.cn (L.H.); 3 Case Comprehensive Cancer Center, Department of Pharmacology, School of Medicine, Case Western Reserve University, Cleveland, OH 44106, USA; youwei.zhang@case.edu

**Keywords:** deep-sea derived fungus, secondary metabolites, ophiobolin, anti-inflammatory effect

## Abstract

Deep-sea fungi have become a new arsenal for the discovery of leading compounds. Here five new ophiobolins **1**–**5**, together with six known analogues **6**–**11**, obtained from a deep-sea derived fungus WHU0154. Their structures were determined by analyses of IR, HR-ESI-MS, and NMR spectra, along with experimental and calculated electronic circular dichroism (ECD) analysis. Pharmacological studies showed that compounds **4** and **6** exhibited obvious inhibitory effects on nitric oxide (NO) production induced by lipopolysaccharide (LPS) in murine macrophage RAW264.7 cells. Mechanical study revealed that compound **6** could inhibit the inducible nitric oxide synthase (iNOS) level in LPS-stimulated RAW264.7 cells. In addition, compounds **6**, **9**, and **10** could significantly inhibit the expression of cyclooxygenase 2 (COX 2) in LPS-induced RAW264.7 cells. Preliminary structure-activity relationship (SAR) analyses revealed that the aldehyde group at C-21 and the α, β-unsaturated ketone functionality at A ring in ophiobolins were vital for their anti-inflammatory effects. Together, the results demonstrated that ophiobolins, especially for compound **6**, exhibited strong anti-inflammatory effects and shed light on the discovery of ophiobolins as new anti-inflammatory agents.

## 1. Introduction

Deep-sea fungi, living under extreme environmental conditions such as high salinity, intensely high pressure, absence of sunlight, and deficiency of nutrients, are considered to be a new reservoir for drug discovery. Recently, a multitude of structurally unique and diverse natural products with promising pharmacological activities have been discovered from deep-sea fungi, which has attracted more attention of researchers for exploring new lead compounds from this type of extreme-environment microorganism [1,2,3,4,5]. Ophiobolins are a group of sesterterpene compounds which are characterized by an intricate 5-8-5 fused ring system. Since Orsenigo isolated ophiobolin A from *Bipolaris oryzae* as a phytotoxin in 1957, around 70 ophiobolin analogues have been discovered from different fungi up to now [6,7,8,9,10,11,12]. Ophiobolins showed a broad spectrum of biological activities including anti-tumor [7,10,11,13], antimicrobial activity [14], anti-inflammatory [12], and calmodulin inhibitory effects [15]. Ophiobolins have attracted more attention of synthetic chemists and pharmacologists due to their structurally and biologically diversity, which show great promising for the development new agents for human diseases treatment [16].

As part of our ongoing efforts to search for new bioactive metabolites from marine microorganisms [17,18], we cultured a nonsporulating deep-sea derived fungus WHU0154. Eleven ophiobolins (compounds **1**–**11**), including five new analogues **1**–**5**, were obtained from the ethyl acetate (EtOAc) extract of the fungus WHU0154. Here we report the isolation and structural elucidation of the new compounds. Meanwhile, the anti-inflammatory activities of these isolated compounds were evaluated and the structure-activity relationship (SAR) was discussed.

## 2. Results

**Figure 1:**
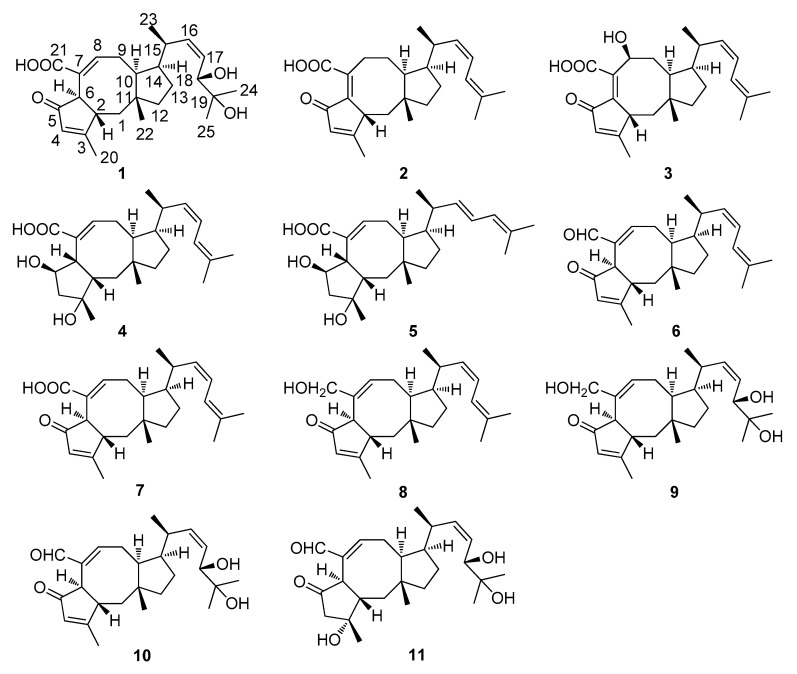
Structures of ophiobolins **1–11** from the fungus strain WHU0154.

The EtOAc extract of fermentation cultures of the fungus strain WHU0154 was subjected to various chromatographic methods, to yield five new and six known ophiobolin-type sesterterpenoids (Figure 1). Based on comparison of MS, NMR, and specific rotation data with those reported in the corresponding literature, the known compounds were identified as 6-epi-ophiobolin G (**6**) [7], asperophiobolin E (**7**) [12], (6α)-21,21-O-dihydroophiobolin G (**8**) [8], (6α)-18,19,21,21-O-tetrahydro-18,19-dihydroxyophiobolin G (**9**) [8], asperophiobolins G (**10**) [12] and (6α)-18,19-O-dihydroxyophiobolin K (**11**) [12], respectively.

### 2.1. Structure Elucidation

**Figure 2:**
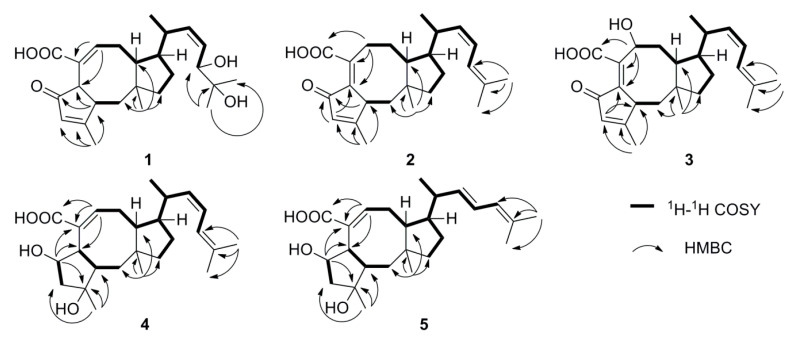
Key ^1^H-^1^H COSY and HMBC correlations of new compounds **1**–**5**.

Compound **1** was obtained as a white amorphous powder. The HR ESI-Q-TOF MS spectrum displayed a pseudomolecular ion at m/z 417.2645 [M + H] ^+^, corresponding to the molecular formula of C_25_H_36_O_5_ (calcd. 417.2641), indicating eight degrees of unsaturation. The ^1^H-NMR spectrum showed the resonances for five methyl protons at δ_H_ 2.11 (3H, s), δ_H_ 1.19 (3H, s), δ_H_ 1.17 (3H, s), δ_H_ 0.99 (3H, s) and δ_H_ 0.99 (3H, d, *J* = 6.0 Hz) and four olefinic protons at δ_H_ 7.00 (1H, s), δ_H_ 5.93 (1H, s), δ_H_ 5.48 (1H, m) and δ_H_ 5.41 (1H, m). The ^13^C-NMR data along with the DEPT 135 spectrum of **1** displayed a total of 25 carbon signals including two carbonyl carbons at δ_C_ 211.6 and 172.0, two oxygenated carbons at δ_C_ 75.3 and 73.6, two olefinic carbons at δ_C_ 181.7 and 130.6, and five methyl carbons at δ_C_ 26.2, 24.9, 23.1, 21.5 and 17.3. Five indices of hydrogen deficiency are attributed to three double bonds and two carbonyls, and the remaining indices of hydrogen deficiency required three additional rings. The ^1^H- and ^13^C-NMR data of **1** closely resembled those of asperophiobolin G (**10**) [12], with the presence of a carbonyl signal at δ_C_ 172.0 in **1** instead of an aldehyde signal at δ_C/H_ 194.7/9.24 seen in **10**. The key HMBC correlations of H-8 (δ_H_ 7.00) to C-21 (δ_C_ 172.0), C-6 (δ_C_ 52.9) and C-7 (δ_C_ 130.6) confirmed that the aldehyde in asperophiobolin G (**10**) was oxidized to a carboxylic acid in **1**. The key ^1^H-^1^H COSY and HMBC correlations confirmed the planar structure of **1** (Figure 2).

**Figure 3:**
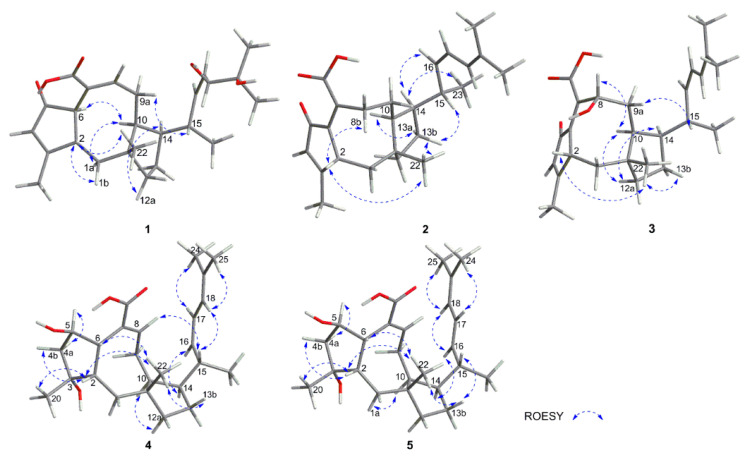
Key ROESY correlations of new compounds **1**–**5**.

**Figure 4:**
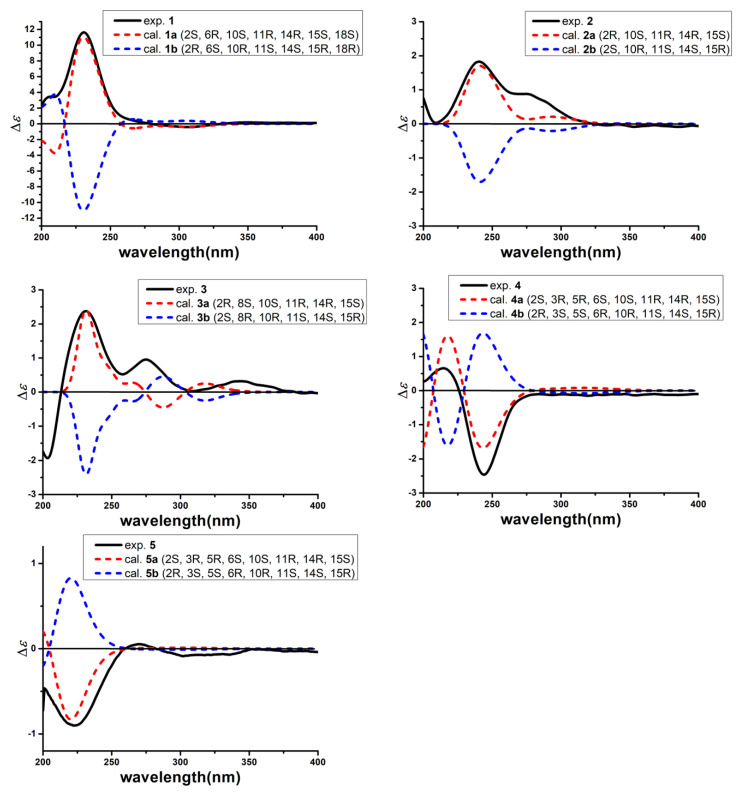
Experimental and calculated electronic circular dichroism (ECD) spectra of compounds **1**–**5**.

The relative configuration of **1** was deduced from a ROESY experiment (Figure 3) as well as by comparing the NMR data with that of **10**. The ROESY correlations of H-2 (δ_H_ 2.88) with H_3_-22 (δ_H_ 0.99), H-1β (δ_H_ 2.16) and of H-6 (δ_H_ 3.44) with H-1α (δ_H_ 1.20) indicated the trans-fused A/B ring which was confirmed by the ^13^C resonance of C-1 at δ_C_ 47.1 and C-22 at δ_C_ 23.1 since the ^13^C resonance signals of C-1 and C-22 usually resonate at higher field when the A/B ring are cis rather than trans [7]. The ROESY correlations of H-1α (δ_H_ 1.20) with H-6 (δ_H_ 3.44), H-10 (δ_H_ 2.65) and H-14 (δ_H_ 1.95), along with of H-1β (δ_H_ 2.16) with H_3_-22 (δ_H_ 0.99) indicated that B/C ring was also trans-fused and H-14 was α-oriented. In addition, the correlations of H-14 (δ_H_ 1.95) with H-15 (δ_H_ 2.64) in the ROESY spectrum revealed that H-15 should be also α-oriented. The ROESY correlation of H-15 (δ_H_ 2.64) and H-18 (δ_H_ 4.17), along with the relatively small *J*_16,17_ value (9.1 Hz), supported the Z-configuration of the Δ^16,17^-double bond. The absolute configuration of the 18,19-diol moiety was identified by the induced ECD spectrum of its in situ complex with Mo_2_(OAc)_4_ in DMSO solution [12,19]. On the basis of the empirical helicity rule relating the sign of the Cotton effect of the diagnostic O-C-C-O moiety, the negative Cotton effect at 310 nm indicated an 18R configuration. Finally, the absolute configuration of compound **1** was determined by experimental and calculated electronic circular dichroism (ECD) analysis (Figure 4). Thus, the structure of compound **1** was determined and named 18,19-dihydro-18,19-dihydroxyasperophiobolin E. Full assignments of the ^1^H- and ^13^C-NMR data were achieved by analyses of 1D- and 2D-NMR spectra (Table 1, Supplementary Figure S1–S10).

Compound **2** was obtained as a white amorphous powder. The molecular formula was deduced as C_25_H_34_O_3_ on the basis of HR ESI-Q-TOF MS and ^13^C-NMR data, indicating nine degrees of unsaturation. The ^1^H-NMR spectrum provided the resonances for five methyl groups and four olefinic protons, while the ^13^C-NMR spectrum exhibited two carbonyl carbons for keto at δ_C_ 196.7 and carboxylic acid group at δ_C_ 174.7 and eight olefinic carbons for four double bonds. Comparison of the NMR data with those of asperophiobolin E (**7**) [12] revealed that they had similar structures, establishing an ophiobolin-based sesterterpenoid nucleus. The double bond at C-6 and C-7 in **2** was identified by HMBC correlations from H-2 (δ_H_ 3.37) to C-6 (δ_C_ 140.8) and from H-8 (δ_H_ 2.52, 2.40) to C-6 (δ_C_ 140.8), C-7 (δ_C_ 139.5) and C-21 (δ_C_ 174.7). Thus, the planar structure of **2** was determined, which was confirmed by key ^1^H-^1^H COSY and HMBC correlations (Figure 2). The relative configuration of compound **2** was determined by ROESY experiments. In the ROESY spectrum, H-2 (δ_H_ 3.37) correlated with H-22 (δ_H_ 1.20), indicating that they were in the same orientation; while H-14 (δ_H_ 1.94) correlated with H-10 (δ_H_ 2.07) and H-15 (δ_H_ 2.64), indicating that they had the same orientation. This demonstrated that the B/C ring was trans-fused while the C-14 side chain was β*-* oriented. Moreover, the coupling constant between H-16 and H-17 (*J*_16,17_ = 9.6 Hz) and ROESY correlation of H-15 (δ_H_ 2.64)/H-18 (δ_H_ 6.06) supported the *Z*-configuration of the Δ^16,17^-double bond. The absolute configuration of compound **2** was determined by experimental and calculated electronic circular dichroism (ECD) analysis (Figure 4). Thus, the structure of compound **2** was identified and named Δ^16,17^-8-dehydroxy-ophiobolin D. Full assignments of the ^1^H- and ^13^C-NMR data were achieved by analyses of 1D- and 2D-NMR spectra (Table 1, Supplementary Figure S11–S20).

Compound **3** was obtained as a white amorphous powder and had a molecular formula of C_25_H_34_O_4_, as determined by the HR ESI-Q-TOF MS and ^13^C-NMR data, requiring nine indices of hydrogen deficiency. The ^1^H- and ^13^C-NMR spectra showed the characteristic signals of ophiobolin sesterterpenoid: five methyl groups at δ_H/C_ 1.40/ 25.5, δ_H/C_ 1.19/26.3, δ_H/C_ 1.17/ 24.9, δ_H/C_ 1.01/ 21.5, and δ_H/C_ 0.90/ 23.7, eight olefinic carbons for four double bonds, and two carbonyl carbons for keto at δ_C_ 198.0 and carboxylic acid group at δ_C_ 175.0. Comparison of the ^1^H- and ^13^C-NMR data with those of **2** suggested that they had the similar structures and the difference between them lay in the chemical shift of C-8. The ^13^C resonance of C-8 at δ_C_ 73.4 in **3**, but not δ_C_ 32.3 in **2**, indicated that compound **3** was a hydroxylated product of **2**. The key ^1^H-^1^H COSY and HMBC correlations confirmed the deduction mentioned above (Figure 2). The relative configuration of compound **3** was identified by analysis of ROESY data and comparison of the NMR data with that of **2** (Figure 3). The key ROESY correlation between H-8 (δ_H_ 4.39) and H-10 (δ_H_ 1.91) indicated that the hydroxyl group of C-8 was β orientation in **3**. The *Z*-configuration of Δ^16,17^ was identified by the ROESY correlation of H-15 (δ_H_ 2.64) and H-18 (δ_H_ 6.09). The absolute configuration of compound **3** was determined by experimental and calculated electronic circular dichroism (ECD) analysis (Figure 4). Thus, the structure of compound **3** was identified and named Δ^16,17^-ophiobolin D. Full assignments of the ^1^H- and ^13^C-NMR data were achieved by analyses of 1D- and 2D-NMR spectra (Table 1, Supplementary Figure S21–S30).

Compound **4** was isolated as a white amorphous powder. It had the molecular formula of C_25_H_38_O_8_ based on analyses of the HR ESI-Q-TOF MS and ^13^C-NMR data, indicating seven degrees of unsaturation. The ^1^H- and ^13^C-NMR spectra showed great similarity to those of ophiobolin U [9], suggested that they had the similar structures. The difference between them was found in the NMR data of C-21. The ^1^H and ^13^C resonance of C-21 changed from δ 9.24/194.7 in ophiobolin U to δ_C_ 174.7 in **4**, indicating that the aldehyde group at C-21 in ophiobolin U was oxidized to carboxylic acid in **4**. Thus, the planar structure of compound **4** was determined, which was confirmed by key ^1^H-^1^H COSY and HMBC correlations (Figure 2). The cis-fused A/B ring system was established by the key ROESY correlation of H-2 (δ_H_ 2.31) with H-6 (δ_H_ 3.17) and the ^13^C resonance of C-1 at δ_C_ 36.7 and C-22 at δ_C_ 19.1 [7]. The other key ROESY correlations in compound **4** matched with those of ophiobolin U, indicating the same stereochemistry. The absolute configuration of compound **4** was determined by experimental and calculated electronic circular dichroism (ECD) analysis (Figure 4). Thus, the structure of compound **4** was identified and named asperophiobolin L. Full assignments of the ^1^H- and ^13^C-NMR data were achieved by analyses of 1D- and 2D-NMR spectra (Table 1, Supplementary Figure S31–S40).

Compound **5** were isolated as a white amorphous powder. It had a molecular formula of C_25_H_38_O_8_ based on the HR ESI-Q-TOF MS and ^13^C-NMR data, the same as that of **4**. The ^1^H- and ^13^C-NMR data of compound **5** was almost identical with those of **4**. The planar structure of compound **5** was confirmed by ^1^H-^1^H COSY and HMBC correlations (Figure 2). Detailed examination of the NMR data revealed that the difference between them lay in the side chain configuration of the double bond. The ROESY correlations of H-17 (δ_H_ 6.03)/H-24 (δ_H_ 1.72) and H-18 (δ_H_ 6.03)/H-25 (δ_H_ 1.79) supported the *Z*-configuration of the Δ^16,17^-double bond in compound **4**; while the *E*-configuration of the Δ^16,17^-double bond in compound **5** can be deduced by the key ROESY correlations of H-16 (δ_H_ 5.22) /H-17 (δ_H_ 6.03) /H-25 (δ_H_ 1.79) and of H-18 (δ_H_ 6.03) /H-24 (δ_H_ 1.72) (Figure 3). The absolute configuration of compound **5** was determined by experimental and calculated electronic circular dichroism (ECD) analysis (Figure 4). Thus, the structure of compound **5** was identified and named (16*E*)-asperophiobolin L. Full assignments of the ^1^H- and ^13^C-NMR data were achieved by analyses of 1D- and 2D-NMR spectra (Table 1, Supplementary Figure S41–S50).

### 2.2. Anti-Inflammatory Bioactivities

**Figure 5:**
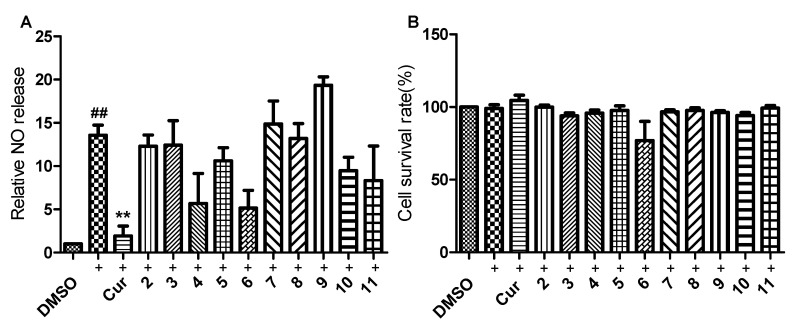
Effects of ophiobolins **2**–**11** on NO production induced by LPS (**A**) and their cytotoxicity (**B**) in murine macrophage RAW 264.7 cells. Cells were pretreated with LPS (500 ng/mL) and then treated with compounds or curcumin at a concentration of 10 µM for 24 h. The data was presented as means ± SEM of three independent experiments. ^##^ *p* < 0.01 vs. control group; ** *p* < 0.01 vs. LPS group.

The anti-inflammatory effects of ophiobolins **2**–**11** were first evaluated by detecting the NO production induced by LPS in murine macrophage RAW264.7 cells. Curcumin was used as positive control. Results showed that compounds **4** and **6** exhibited obvious inhibitory effects on NO production induced by LPS in RAW264.7 cells although no statistical significance was observed (Figure 5A). Meanwhile, these compounds except **6** had no cytotoxicity at all toward RAW264.7 cells at indicated concentrations (Figure 5B). Even though compound **6** reduced the cell survival rate to ~77 ± 18%, there was no statistical difference between control and compound **6** (Figure 5B). Therefore, these compounds did not significantly affect cell survival.

**Figure 6:**
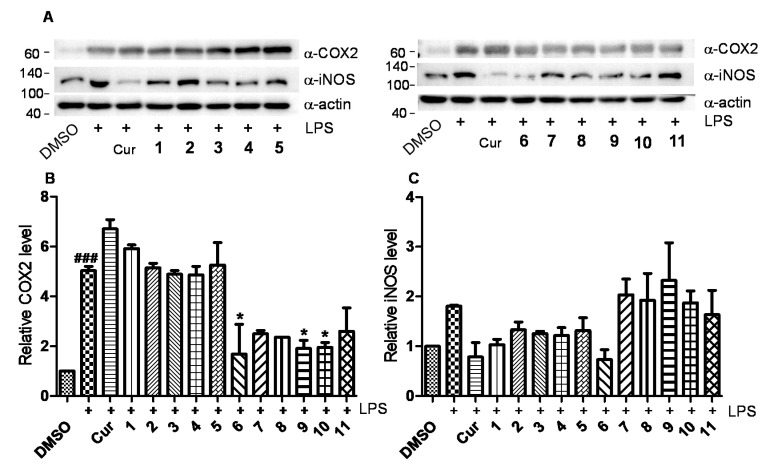
Effect of ophiobolins **1**–**11** on COX 2 and iNOS protein levels in LPS-stimulated murine macrophage RAW 264.7 cells. Cells were pretreated with LPS (100 ng/mL) and then treated with compounds (10 µM) or curcumin (20 µM) for 24 h. The total protein levels were analyzed by western blot. (**A**) The expression of COX 2 and iNOS protein levels. (**B**) The quantification histogram of COX 2 and iNOS (**C**). ^###^ *p* < 0.001 vs. control group; * *p* < 0.05 vs.LPS group.

Mechanical sudies revealed that compound **6** could obviously inhibit the expression of iNOS induced by LPS in RAW264.7 cells (Figure 6A–C). Also, compounds **6**, **9**, and **10** could inhibit the expression of COX 2 induced by LPS in RAW264.7 cells (Figure 6A,B). Preliminary structure-activity relationship (SAR) analysis revealed that the aldehyde group at C21 in ophiobolins is vital for their anti-inflammatory activities since compounds **1**–**3** and **7** didn’t show obvious bioactivities. Also, α,β-unsaturated ketone in A ring may also be crucial for their anti-inflammatory effects since compound **11** didn’t exhibit obvious anti-inflammatory effect.

Together, our studies demonstrated that ophiobolin **6** could inhibit the NO production induced by LPS in RAW264.7 cells by suppressing the expression of iNOS. Also, compounds **6**, **9**, and **10** could exhibit anti-inflammatory effect by inhibiting the expression of COX 2. These results have broaden the application of ophiobolins.

## 3. Discussion

Ophiobolins are widely distributed in fungus secondary metabolites, produced by the pathogenic plant fungi [6,20,21], mangrove endophytic fungi [12,22], and fungi from marine sediments [7,21]. Here we first reported the discovery of ophiobolins from deep-sea fungus from around 3200 m depth of the South China Sea. Previous studies were mainly focus on the phytotoxin, anti-bacterial and anticancer activities of this kind of compounds [7,10,11,13,14,20,21,22,23]. Scattered reports were involved in the anti-inflammatory effects of this type of compounds [12,24]. Aniko et al., reported that treatment of male Wistar rats with 1.0 mg/kg of ophiobolin A could promote systemic inflammation by elevating the concentration of interleukin-6 (IL-6) and tumor necrosis factor-alpha (TNF-α) as well as the activity of heme oxygenase (HO) and myeloperoxidase (MPO) in plasma [24]. Cai et al., reported that some ophiobolins could inhibit the production of NO induced by LPS in RAW264.7 macrophage cells [12]. Our results were line with that reported by cai et al., The contradictory results may be explained by different models of assessment and treatment dose and time point.

In addition, here we first discussed the preliminary SAR analysis of the anti-inflammatory effects of ophiobolins and their underlying mechanisms. Combined with previous report [12], we can conclude that the aldehyde group at C-21 and the α,β-unsaturated ketone functionality at A ring in ophiobolins were indeed crucial for their anti-inflammatory effects. In addition, compound **6** only slightly affected the cell survive with no statistical significance was observed, which indicated us to assess its cytotoxicity while evaluating its anti-inflammatory. To sum up, our results provide critical information for further medicinal chemistry research of ophiobolins and help for the discovery of more potent anti-inflammatory agents.

## 4. Materials and Methods

### 4.1. General Experimental Procedure

1D- and 2D-NMR data were determined with a Bruker AV 600 instrument (Bruker, Bremen, Germany) using solvent signal (CD_3_OD: δ_H_ 3.31/δ_C_ 49.0) as an internal reference. A JASCO FT/IR-480 plus spectrometer (JASCO International Co. Ltd., Tokyo, Japan) was used to measure the IR spectra. The UV/vis spectra were obtained using a JASCO V-550 UV/Vis (JASCO International Co. Ltd., Tokyo, Japan) and HR-ESI-MS spectra were acquired on a Waters Synapt G2 mass spectrometer (Waters, Manchester, UK). HPLC analyses were carried out on an LC-20AB Liquid Chromatography system equipped with a SPD-M20A DAD detector (Shimadzu, Kyoto, Japan) using a Cosmosil-Triart C_18_ column (5 µm, φ 4.6 × 250 mm, NacalaiTesque, Kyoto, Japan). The semi-preparative HPLC was performed on a Shimadzu LC-20AT Liquid Chromatography system (Shimadzu, Kyoto, Japan) with a SPD-20A UV/Vis detector (Shimadzu, Kyoto, Japan) using a Cosmosil-Pack ODS-A column (5 μm, φ 10 × 250 mm, NacalaiTesque, Kyoto, Japan).

MeOH and acetonitrile (CH_3_CN) for HPLC were purchased from BCR International Co. Ltd. (Shanghai, China) and Merck (Darmstadt, Germany), respectively. Silica gel (200–300 mesh, Qingdao Marine Chemical Ltd., Shandong, China), and octadecylsilanized (ODS) (12 nm, 50 μm, YMC Ltd., Tokyo, Japan) were used for column chromatography (CC). Pre-coated silica gel plates (SGF_254_, 0.2 mm, Yantai Chemical Industry Research Institute, Shandong, China) were used for the TLC analyses.

DMSO and methyl thiazolyl tetrazolium (MTT) were purchased from Aladdin Reagent Co., Ltd. (Shanghai, China). NO assay kit was purchased from Beyotime Institute of Biotechnology (Shanghai, China). Antibodies for iNOS (#13120S) and COX 2 (#12282T) were purchased from Cell Signaling Technology (Beverly, MA, USA). Antibody for β-action (#AP0060) was purchased from Bioworld Technology Inc (Louis Park, MN, USA). HRP-conjugated secondary anti-rabbit (#GTX213110-01) and anti-mouse (#GTX213111-01) antibodies were acquired from GeneTex Inc (Irvine, CA, USA).

### 4.2. Fungal Material and Identification

Strain WHU0154 was isolated from a deep sea sample at 117°51.41′E, 19°50.71′N and 3197 m depth, South China Sea, on Thayer-Martin agar (glucose 10 g, peptone 5 g, KH_2_PO_4_ 1 g, MgSO_4_ 0.5 g, Rose Bengal 0.03 g, sea salts 15 g, agar 20 g, ddH_2_O 1L). The strain was first selected by antibacterial activities against *Staphylococcus aureus* ATCC 5165 of its culture crude extracts. It was identified as *Aspergillus sp.* according to the morphological characteristics of typical Aspergillus conidia structure (Supplementary Figure S51) and the internal transcribed spacer (ITS) sequence (GeneBank accession number MW228045), which is 98.85% similarity to *Aspergillus asper* NRRL 35910 (NCBI Reference Sequence: NR_151788.1), and is stored at −80 °C in School of Pharmaceutical Sciences, Wuhan University, China.

### 4.3. Cultivation, Extraction and Isolation

The initial cultures were maintained on the potato dextrose agar PDA (200 g of potato, 20 g of glucose, 20 g of agar, 1 L of pure water, pH natural) solid medium plates for 3 or 4 days. Matured spores were aseptically inoculated into 15 conical flasks (500 mL), each containing one-third of the PDA liquid medium (200 g of potato, 20 g of glucose, 1 L of pure water, pH natural), and continued to be cultured as seed solution for 7 days. Then the seed solution was aseptically inoculated into 500 mL/1L/3L conical flasks, each containing one-third of the liquid medium, the fermentation was performed on PDA liquid medium and statically cultured at 25 °C for 27 days.

### 4.4. Extraction and Isolation

The whole fermentation broth (16 L) was filtered by Buchner filter to separate the mycelia and the filtrate. The filtrate was extracted with an equal volume of ethyl acetate (EtOAc) for three times. The combined organic layer was concentrated under reduced pressure to afford 3.7 g of extract. The EtOAc extract (3.7 g) was subjected to silica gel (200–300 mesh) column chromatography using a petroleum ether-EtOAc gradient (from 1: 0 to 0: 1) and EtOAc-MeOH (0: 1) to obtain 10 fractions (Fr.1 to Fr.10). Fraction 6 (0.53 g) was applied to an open ODS column chromatography eluted with stepwise gradient of 20–100% MeOH-H_2_O to get ten subfractions (Fr.6-1 to Fr.6-10). The subfraction 6-9 (0.2528 g) was further chromatographed by a silica gel (200–300 mesh) column chromatography using a Petroleum ether-EtOAc (95:5/9:1/8:2/1:1/0:1) and EtOAc-MeOH (0: 1) to give six subfractions (Fr.6-9-1 to Fr.6-9-6). The subfraction 6-9-2 (32.6 mg) was purified by semipreparative reversed-phase (RP) HPLC [65% MeCN-H_2_O (0.1% CH_3_COOH)] to yield compounds **2** (1.7 mg) and **6** (2.6 mg). The subfraction 6–10 (58.5 mg) was subjected to RP-HPLC (80% MeOH-H_2_O) to obtain compounds **7** (1.9 mg) and **8** (1.6 mg). The fraction 7 (1.20 g) was subjected to an ODS column chromatography by stepwise gradient elution of 20−100% MeOH-H_2_O to get 10 subfractions (Fr.7-1 to Fr.7-10). The subfraction 7–5 (127.0 mg) was purified by semipreparative RP-HPLC [28% MeCN-H_2_O (0.1% CH_3_COOH)] to obtain compound **10** (13.1 mg). The subfraction 7–6 (0.56 g) was applied to silica gel CC eluted with gradient Petroleum ether-EtOAc (9: 1/8: 2/7: 3/1: 1/0: 1) and EtOAc-MeOH (0: 1) to get seven subfractions (Fr.7-6-1 to Fr.7-6-7). The subfraction 7-6-4 (111.5 mg) was purified by RP-HPLC [45% MeOH-H_2_O (0.1% CH_3_COOH)] to obtain compound **11** (4.5 mg). The subfraction 7-6-5 (82.6 mg) was subjected to RP-HPLC (65% MeOH-H_2_O) to obtain compounds **1** (2.4 mg) and **9** (3.3 mg). The subfraction 7–8 was subjected to RP-HPLC eluted with 80% MeOH-H_2_O to afford compounds **3** (5.3 mg), **4** (25.1 mg), and **5** (2.6 mg).

*18,19-Dihydro-18,19-dihydroxyasperophiobolin E* (**1**): White amorphous powder; [α${]}_{D}^{35}$ + 70.7 (*c* 1.3, MeOH); UV(MeOH)(logε) λ_max_ 230(2.59); IR (KBr) *ν*_max_ 3434, 2965, 2927, 2666, 1681, 1617, 1553, 1455, 1379, 1316, 1261, 1220, 1187, 1156, 1049, 1017, 962, 904, 858, 797, 756 cm^−1^; CD (MeOH) (∆ε) 306 (−0.42), 230 (+11.6); ^1^H- and ^13^C-NMR data, see Table 1; HRESIMS *m/z* 417.2645 [M + H]^+^ (calcd for C_25_H_37_O_5_, 417.2641).

*Δ^16,17^-8-Dehydroxyophiobolin* D (**2**): White amorphous powder; [α${]}_{D}^{35}$ + 77.1 (*c* 0.45, MeOH); UV(MeOH)(logε) λ_max_ 241(1.43); IR (KBr) *ν*_max_ 2965, 2863, 2730, 1605, 1455, 1385, 1127, 1080 cm^−1^; CD (MeOH) (∆ε) 283 (+0.72), 242 (+1.73), 208(−0.89); ^1^H- and ^13^C-NMR data, see Table 1; HRESIMS *m/z* 383.2595 [M + H]^+^ (calcd for C_25_H_34_O_3_, 383.2586).

*Δ^16,17^-Ophiobolin D* (**3**): White amorphous powder; [α${]}_{D}^{35}$+ 112.5 (*c* 2.4, MeOH); UV (MeOH) (logε) λ_max_ 241(2.81); IR (KBr) *ν*_max_ 3428, 2930, 1687, 1608, 1382, 1135 cm^−1^; CD (MeOH) (∆ε) 351 (+0.39), 275 (+0.99), 233(+1.87), 205(−4.4); ^1^H- and ^13^C-NMR data, see Table 1; HRESIMS *m/z* 399.2537 [M + H]^+^ (calcd for C_25_H_35_O_4_, 399.2535).

*Asperophiobolin L* (**4**): White amorphous powder; [α${]}_{D}^{35}$ + 24.5 (*c* 1.7, MeOH); UV(MeOH)(logε) λ_max_ 238(2.87); IR (KBr) *ν*_max_ 2944, 1643, 1452, 1376, 1267, 1194, 1098, 1057, 666 cm^−1^; CD (MeOH) (∆ε) 244 (−3.45), 218 (−0.96), 207 (−1.74); ^1^H- and ^13^C-NMR data, see Table 1; HRESIMS *m/z* 805.5614 [2M + H]^+^ (calcd for C_50_H_77_O_8_, 805.5618).

*(16E)-Asperophiobolin L* (**5**): White amorphous powder; [α${]}_{D}^{35}$ + 7.6 (c 1.95, MeOH); UV (MeOH) (logε) λ_max_ 221(1.75); IR (KBr) *ν*_max_ 2962, 2857, 2736, 1678, 1631, 1449, 1385, 1200, 1141 cm^−1^; CD (MeOH) (∆ε) 302(−0.08), 270 (−0.05), 223 (−0.9); ^1^H- and ^13^C-NMR data, see Table 1; HRESIMS *m/z* 805.5621 [2M + H]^+^ (calcd for C_50_H_77_O_8_, 805.5618).

### 4.5. Quantum Chemical ECD Calculations of 1–5

The electronic circular dichroism (ECD) spectra of each conformer for **1**–**5** were calculated by the TDDFT methodology with MeOH as solvent. Firstly, the SMILES codes of molecules of **1a** (2*S*, 6*R*, 10*S*, 11*R*, 14*R*, 15*S*, 18*S*), **2a** (2*R*, 10*S*, 11*R*, 14*R*, 15*S*), **3a** (2*R*, 8*S*, 10*S*, 11*R*, 14*R*, 15*S*), **4a** (2*S*, 3*R*, 5*R*, 6*S*, 10*S*, 11*R*, 14*R*, 15*S*) and **5a** (2*S*, 3*R*, 5*R*, 6*S*, 10S, 11*R*, 14*R*, 15*S*) were acquired before the initial 3D structures were generated with CORINA version 3.4 (https://www.mn-am.com/online_demos/corina_demo). Subsequently, CONFLEX version 7.0 (http://www.conflex.net/) was used to acquire the conformer databases via the MMFF94s force-field, during which, an energy window of 5 kcal mol^−1^ for **1**–**4** and 3 kcal mol^−^^1^ for **5**, above the ground state, for acceptable conformers (ewindow), a maximum number of conformations per molecule (maxconfs) of 100, and an RMSD cutoff (rmsd) of 0.5Å were considered. Then, all the acceptable conformers were optimized with HF/6-31G(d) method in Gaussian 09, respectively, and subsequent further optimization at the B3LYP/6-31G(d) level with MeOH led the dihedral angles to be got. After that, stable conformers, 74 for **1a**, 9 for **2a**, 23 for **3a**, 48 for **4a** and 42 for **5a**, were obtained. The optimized conformers were taken for the ECD calculations by Gaussian 09 at the B3LYP/TZVP level. The solvent effect was taken into account by the polarizable-conductor calculation model (IEFPCM, MeOH as the solvent) [25]. Then, the electronic circular dichroism (ECD) spectra of **1b** (2*R*, 6*S*, 10*R*, 11*S*, 14*S*, 15*R*, 18*R*), **2b** (2*S*, 10*R*, 11*S*, 14*S*, 15*R*), **3b** (2*S*, 8*R*, 10*R*, 11*S*, 14*S*, 15*R*), **4b** (2*R*, 3*S*, 5*S*, 6*R*, 10*R*, 11*S*, 14*S*, 15*R*) and **5b** (2*R*, 3*S*, 5*S*, 6*R*, 10*R*, 11*S*, 14*S*, 15*R*) were also calculated as the same method. Finally, the software SpecDis (Version 1.70, Berlin, Germany). was used to compare the experimental and calculated spectra of **1**–**5**, during which, a UV shift to the ECD spectra, Gaussian broadening of the excitations, and Boltzmann weighting of the spectra were considered [26].

### 4.6. Cell Culture

The murine macrophage RAW264.7 cells were obtained from the American Type Culture Collection (ATCC, Rockefeller, MD, USA). The cells were grown in Dulbecco’s modified Eagle’s medium (DMEM) medium with 10% FBS (ExCell bio, Shanghai, China) at 37 °C in a 98% humidified incubator with 5% CO_2_ and 95% air. The cells were routinely split once every 1–2 days.

### 4.7. Measurement of Cell Viability

MTT assay was used to determine the effects of ophiobolins on the viability of RAW264.7 cells. All compounds were dissolved in DMSO at 10 mM stock concentration and stored at −20 °C. DMSO was used as blank control. The final concentration of DMSO kept below 0.1% in cell culture throughout the biological study. The cells were seeded in a 96-well plate (1.5 × 10^4^ cells/well) overnight and treated with compounds (10 μM) and LPS (500 ng/mL) for 24 h. Then, 20 μL of MTT (5 mg/mL) was added to each well for an additional 4 h. The resulting formazan crystals after aspiration of the culture medium were dissolved in DMSO (150 μL/well) and the optical densities (OD, Synergy HT, BioTek, VT, USA) were measured at 570 nm. The data was presented as means ± SEM of three independent experiments.

### 4.8. NO Assay

The Griess method was used to measure NO concentrations in culture supernatants. Cells were seeded into 96-well plates at a density of 1.5 × 10^4^ cells/well. After adhesion, the cells were treated with 10 μM of compounds and LPS (500 ng/mL) for 24 h. Nitrite release in the culture media was determined using the Griess reaction and presumed to reflect the NO levels. Briefly, the samples were mixed with equal volume of Griess reagent (1% sulfanilamide in 5% phosphoric acid and 0.1% naphthylethylenediamine dihydrochloride) and then incubated at room temperature for 10 min. The absorbance was measured at 540 nm on a microplate reader (Synergy HT, BioTek, VT, USA). And the NO concentration was determined at 540 nm using NaNO_2_ as a standard. The data was presented as means ± SEM of three independent experiments

### 4.9. Western Blot

Briefly, 2 × 10^6^ cells/well were seeded in a 6-well flat-bottomed plate, grown at 37 °C for 24 h, and treated with ophiobolin derivatives(10 μM) and LPS (100 ng/mL) for 24 h. Total cell lysates were harvested in lysis buffer (Beyotime Inst. Biotech, Shanghai, China) containing 1 mM phenylmethylsulfonyl fluoride (Beyotime Inst. Biotech). The protein concentration was measured using the Pierce^®^ BCA Protein Assay Kit (#23225, Pierce, Thermo, MA, USA). Equal amount of total proteins (~40 μg) were separated on 6–15% polyacrylamide gelelectrophoresis (SDS-PAGE) and transferred to poly(vinylidene fluoride) (PVDF) membranes (#IPVH00010, Millipore, Billerica, MA, USA). The membranes were blocked with 5% skim milk and probed with primary antibodies specific for iNOS, COX 2 and β-actin overnight at 4 °C followed by horseradish peroxidase conjugated secondary antibodies for 1 h, reacted with Pierce^®^ ECL Western Blotting Substrate (Thermo Fisher Scientific, Franklin, MA, USA) and detected by an ECL detection imaging system (BioTanon, Shanghai, China). The data was presented as means ± SEM of three independent experiments

### 4.10. Statistical Analysis

The statistical analyses were performed by using GraphPad Prism software version 5 (GraphPad Software, Inc., San Diego, CA, USA). Each experiment was performed at least three replicates and the results were presented as mean ± SEM. Multiple comparisons were carried out by one-way ANOVA, followed by Tukey’s test. *p* < 0.05 was considered statistically significant.

## Figures and Tables

**Table 1:** ^1^H- and ^13^C-NMR data assignments of ophiobolins **1**-**5** (600 MHz for ^1^H and 150 MHz for ^13^C in CD_3_OD).

	1		2		3		4		5	
	δ_C_	δ_H_ (*J* in Hz)	δ_C_	δ_H_ (*J* in Hz)	δ_C_	δ_H_ (*J* in Hz)	δ_C_	δ_H_ (*J* in Hz)	δ_C_	δ_H_ (*J* in Hz)
1	47.1	2.16(1H,m) 1.20(1H,m)	50.7	2.12(1H,m); 0.99(1H,dd, 15.4, 10.5)	49.8	2.06(1H,d, 12.3); 0.94(1H)	36.7	1.54(1H,dd, 12.1, 8.7); 1.11(1H,t, 13.5)	37	1.58(1H,m) 1.13; (1H,ddd, 16.0, 13.6, 9.0)
2	51.2	2.88(1H,d,12.9)	47.2	3.37(1H,d,12.3)	45.1	3.95(1H,d, 11.1)	51.8	2.31(1H,m)	51.9	2.35(1H,m)
3	181.7		180.9		181.5		82.9		82.9	
4	130.3	5.93(1H,s)	131.5	6.03(1H,m)	130.5	6.06(1H,s)	53.1	2.44(1H,dd,14.9, 7.8); 1.79(1H,m)	53.2	2.46(1H,dd,14.9, 7.8); 1.81(1H,m)
5	211.6		196.7		198		75.1	4.52(1H,m)	75.2	4.55(1H,m)
6	52.9	3.44(1H,d, 3.4)	140.8		138.7		52.3	3.07(1H,d, 10.1)	52.3	3.11(1H,d, 10.1)
7	130.6		139.5		139.7		134		134.4	
8	146.5	7.00(1H,s)	32.3	2.52(1H,td,13.2, 6.1); 2.40 (1H,dd,12.1, 5.1)	73.4	4.39(1H,t,6.5)	147.7	6.88(1H,t,8.8)	147.1	6.89(1H,t,8.7)
9	30.9	3.00(1H,d,19.8) 2.20(1H,m)	26.9	1.58(1H,m) 2.01(1H,m)	32.3	2.24(1H,ddd,15.0, 5.9,3.7); 1.85 (1H,dd,14.9, 7.4)	25.7	2.68(1H,ddd,15.1, 13.5, 5.8) 2.07(1H,m)	26.9	2.53 (1H,dd,12.9, 8.4); 2.09 (1H,dd,11.7,10.3)
10	45.2	2.65(1H,m)	45.2	2.07(1H,m)	45.4	1.91(1H,m)	55.5	1.54(1H,dd,12.1, 8.7)	55.7	1.58(1H,m)
11	46.4		44.9		44.7		44.9		44.8	
12	45.3	1.54(1H,m) 1.48(1H,m)	46.1	1.52(1H,m) 1.38(1H,m)	44.3	1.55(1H,m) 1.29(1H,m)	43.9	1.40(2H,m)	44.1	1.43(2H,dd,9.5, 4.4)
13	28.6	1.68(1H,m) 1.36(1H,m)	29.3	1.59(1H,m) 1.47(1H,m)	29.3	1.68(1H,m) 1.55(1H,m)	27.7	1.60(1H,m) 1.40(1H,m)	27.5	1.58(2H,m)
14	53.2	1.95(1H,m)	52.3	1.94(1H,dd,17.3,8.2)	50.5	2.06(1H,d, 12.3)	48.6	2.07(1H,m)	48.6	2.18(1H,ddd,25.8, 12.2, 6.9)
15	33.8	2.63(1H,m)	34.1	2.64(1H,dd,16.0, 9.2)	35.4	2.64(1H,m)	37.2	2.74(1H,ddd,15.1, 13.5, 5.8)	42.9	2.36(1H,m)
16	141.9	5.48(1H,m)	138.9	5.20(1H,t,9.8)	138.0	5.21(1H,m)	138.7	5.22(1H,m)	140.3	5.47(1H,dd, 15.1, 8.6)
17	127.7	5.41(1H,m)	123.4	6.03(1H,m)	123.8	6.09(1H,m)	123.2	6.03(1H,m)	125.9	6.16(1H,dd, 15.1, 10.8)
18	75.3	4.17(1H,d,9.6)	121.7	6.03(1H,m)	121.6	6.09(1H,m)	121.5	6.03(1H,m)	126.5	5.77(1H,d, 10.7)
19	73.6		135.7		136.2		136		133.3	
20	17.3	2.11(3H,s)	17.6	2.17(3H,s)	17.5	2.17(3H,s)	26.5	1.22(3H,s)	26.5	1.24(3H,s)
21	172		174.7		174.5		174.7		175.2	
22	23.1	0.99(3H,s)	21.9	1.20(3H,s)	22.4	1.19(3H,s)	19.1	0.99(3H,s)	19.3	0.99(3H,s)
23	21.5	0.99(3H,d,6.0)	21.2	0.91(3H,d,6.5)	20.7	0.95(3H,d,6.5)	20.8	0.89(3H,d,6.6)	21	0.96(3H,d,6.6)
24	24.9	1.17(3H,s)	18.1	1.75(3H,s)	18.1	1.75(3H,s)	18.1	1.72(3H,s)	18.2	1.74(3H,s)
25	26.2	1.19(3H,s)	26.5	1.82(3H,s)	26.5	1.82(3H,s)	26.5	1.79(3H,s)	26	1.76(3H,s)

## Supplementary Materials

The HR-ESI-Q-TOF-MS, UV, IR, and 1D-, 2D-NMR spectra of compounds **1**–**5**, along with cultural and microscopic morphology of strain WHU0154, are available online at https://www.mdpi.com/1660-3397/18/11/575/s1.

## References

[1] Arifeen M.Z., Ma Y.N., Xue Y.R., Liu C.H. (2020). Deep-sea fungi could be the new arsenal for bioactive molecules. Mar. Drugs.

[2] Sun C.X., Shah M., Zhang Z.Z., Feng Y.Y., Chang Y.M., Che Q., Gu Q.Q., Zhu T.J., Zhang G.J., Li D.H. (2019). Secondary metabolites from deep-sea derived microorganisms. Curr. Med. Chem..

[3] Skropeta D., Wei L.Q. (2014). Recent advances in deep-sea natural products. Nat. Prod. Rep..

[4] Daletos G., Ebrahim W., Ancheeva E., El-Neketi M., Song W.G., Lin W.H., Proksch P. (2018). Natural products from deep-sea-derived fungi-a new source of novel bioactive compounds?. Curr. Med. Chem..

[5] Ogaki M.B., Coelho L.C., Vieira R., Neto A.A., Zani C.L., Alves T.M.A., Junior P.A.S., Murta S.M.F., Barbosa E.C., Oliveira J.G. (2020). Cultivable fungi present in deep-sea sediments of Antarctica: Taxonomy, diversity, and bioprospecting of bioactive compounds. Extremophiles.

[6] Au T.K., Chick W.S.H., Leung P.C. (2000). The biology of ophiobolins. Life Sci..

[7] Hong W., Takuya I., Masahiro K., Yasuhide N., Mineko K., Motomasa K. (2004). Cytotoxic sesterterpenes, 6-epi-ophiobolin G and 6-epi-ophiobolin N, from marine derived fungus *Emercella variecolor* GF10. Tetrahedron.

[8] Liu H.B., Edrada-Ebel R., Ebel R., Wang Y., Schulz B., Draeger S., Muller W.E.G., Wray V., Lin W.H., Proksch P. (2011). Ophiobolin Sesterterpenoids and Pyrrolidine Alkaloids from the Sponge-Derived Fungus *Aspergillus ustus*. Helv. Chim. Acta.

[9] Bladt T.J., Durr C., Knudsen P.B., Kildgaard S., Frisvad J.C., Gotfredsen C.H., Seiffert M., Larsen T.O. (2013). Bio-activity and dereplication-based discovery of ophiobolins and other fungal secondary metabolites targeting leukemia cells. Molecules.

[10] Zhu T.H., Lu Z.Y., Fan J., Wang L.P., Zhu G.L., Wang Y., Li X., Hong K., Piyachaturawat P., Chairoungdua A. (2018). Ophiobolins from the mangrove fungus *Aspergillus ustus*. J. Nat. Prod..

[11] Choi B.K., Trinh P.T.H., Lee H.S., Choi B.W., Kang J.S., Ngoc N.T.D., Van T.T.T., Shin H.J. (2019). New ophiobolin derivatives from the marine fungus *Aspergillus flocculosus* and their cytotoxicities against cancer cells. Mar. Drugs.

[12] Cai R.L., Jiang H.M., Mo Y.L., Guo H.X., Li C.Y., Long Y.H., Zang Z.M., She Z.G. (2019). Ophiobolin-type sesterterpenoids from the mangrove endophytic fungus *Aspergillus sp*. ZJ-68. J. Nat. Prod..

[13] Zhao Y., Zhao C.X., Lu J., Wu J., Li C.H., Hu Z.Y., Tian W., Yang L., Xiang J., Zhou H.B. (2019). Sesterterpene MHO7 suppresses breast cancer cells as a novel estrogen receptor degrader. Pharmacol. Res..

[14] Li E.G., Clark A.M., Rotella D.P., Hufford C.D. (1995). Microbial metabolites of ophiobolin A and antimicrobial evaluation of ophiobolins. J. Nat. Prod..

[15] Leung P.C., Taylor W.A., Wang J.H., Tipton C.L. (1985). Role of calmodulin inhibition in the mode of action of ophiobolin A. Plant Physiol..

[16] Brill Z.G., Grover H.K., Maimone T.J. (2016). Enantioselective synthesis of an ophiobolin sesterterpene via a programmed radical cascade. Science.

[17] Chen Y.P., Liu Q., Gao H., Lin H.P., Tian H.Y., Hong K., Li J., Jiang R.W., Yao X.S., Tang J.S. (2014). Streptospirodienoic acids A and B, 6,6-spiroketal polyketides from *Streptomyces sp*. RSC Adv..

[18] Gou X.S., Jia J., Xue Y.X., Ding W.J., Dong Z.T., Tian D.M., Chen M., Bi H.K., Hong K., Tang J.S. (2020). New pyrones and their analogs from the marine mangrove-derived *Aspergillus sp*. DM94 with antibacterial activity against *Helicobacter pylori*. Appl. Microbiol. Biotechnol..

[19] Bari L.D., Pescitelli G., Pratelli C., Pini D., Salvadori P. (2001). Determination of absolute configuration of acyclic 1,2-diols with Mo2(OAc)4. 1. Snatzke’s method revisited. J. Org. Chem..

[20] Liu X.H., Miao F.P., Qiao M.F., Cichewicz R.H., Ji N.Y. (2013). Terretonin, ophiobolin, and drimane terpenes with absolute configurations from an algiconus *Aspergillus ustus*. RSC Adv..

[21] Masayoshi A., Hiroki N., Motomasa K. (2013). Marine-derived fungal sesterpenes, ophiobolins, inhibit biofilm formation of *Mycobacterium* species. J. Nat. Med..

[22] Xiao J.Z., Tsuda M., Doke N., Nishimura S. (1991). Phytotoxins produced by germinating spores of Bipolaris oryzae. Phytopathology.

[23] Antonio E., Anna A., Alessio C., Maurizio V., Mariano F., Raghavan C., Andrea M. (2006). Ophiobolin E and 8-epi-ophiobolin J produced by Drechslera gigantean, a potential mycoherbicide of weedy grasses. Phytochemistry.

[24] Zhang J., Zhao S.S., Xie J., Yang J., Chen G.D., Hu D., Zhang W.G., Wang C.X., Yao X.S., Gao H. (2020). N-methoxy-β-carboline alkaloids with inhibitory activities against Aβ42 aggregation and acetylcholinesterase from the stems of Picrasma quassioides. Bioorg. Chem..

[25] Torsten B., Anu S., Yasmin H., Gerhard B. (2013). SpecDics: Quantifying the comparison of calculated and experimental electronic circular dichroism spectra. Chirality.

[26] Pósa A., Szabó R., Szalai Z., Kupai K., Deim Z., Murlasits Z., Bencsik O., Szekeres A., Vágvölgyi C., Laszlo B. (2016). The effect of acute ophiobolin A treatment on HO-mediated inflammatory processes. Hum. Exp. Toxicol..

