# Supplementary material for: New Ophiobolins from the Deep-Sea Derived Fungus Aspergillus sp. WHU0154 and Their Anti-Inflammatory Effects
Source: Mar Drugs. 2020 Nov 20;18(11):575. doi: 10.3390/md18110575 (PMC7699878; doi:10.3390/md18110575)

Supplementary data

New ophiobolins from the deep-sea derived fungus *Aspergillus sp*. WHU0154 and their anti-inflammatory effects

Wenjuan Ding [1,†], Chokkalingam Uvarani [1,†], Fangfang Wang [1,†], Yaxin Xue [2], Ning Wu [2], Liming He [2], Danmei Tian [1], Mei Chen [1], Youwei Zhang [3], Kui Hong [2,*] and Jinshan Tang [1,*]

[1] Institute of Traditional Chinese Medicine and Natural Products, International Cooperative Laboratory of Traditional Chinese Medicine Modernization and Innovative Drug Development of Chinese Ministry of Education (MOE), College of Pharmacy, Jinan University, Guangzhou 510632, China

[2] Key Laboratory of Combinatorial Biosynthesis and Drug Discovery, Ministry of Education, School of Pharmaceutical Sciences, Wuhan University, Wuhan 430071, China

[3] Department of Pharmacology, Case Comprehensive Cancer Center, Case Western Reserve University School of Medicine, Cleveland, OH 44106,USA

[†] These authors contributed equally to this work.

***** Correspondence: jstang0615@jnu.edu.cn; Tel.: +86-20-85221559 (J.T.); kuihong31@whu.edu.com; Tel.: +86-27-68752442 (K.H.)

Fig. S1 HR ESI-Q-TOF-MS spectrum of compound **1**................ 4
Fig. S2 UV spectrum of compound **1**..4
Fig. S3 IR spectrum of compound **1**...4
Fig. S4 ^{1}H-NMR spectrum of compound **1**..5
Fig. S5 ^{13}C-NMR spectrum of compound **1**...5
Fig. S6 DEPT 135 spectrum of compound **1**............................. 6
Fig. S7 ^{1}H-^{1}H COSY spectrum of compound **1**..6
Fig. S8 HSQC spectrum of compound **1**...7
Fig. S9 HMBC spectrum of compound **1**...7
Fig. S10 **ROESY** spectrum of compound **1**..8
Fig. S11 HR ESI-Q-TOF-MS spectrum of compound **2**........................... 8
Fig. S12 UV spectrum of compound **2**..9
Fig. S13 IR spectrum of compound **2**..9
Fig. S14 ^{1}H-NMR spectrum of compound **2**..9
Fig. S15 ^{13}C-NMR spectrum of compound **2**...10
Fig. S16 DEPT 135 spectrum of compound **2**............................ 10
Fig. S17 ^{1}H-^{1}H COSY spectrum of compound **2**...11
Fig. S18 HSQC spectrum of compound **2**..11
Fig. S19 HMBC spectrum of compound **2**...12
Fig. S20 **ROESY** spectrum of compound **2**.. 12
Fig. S21 HR ESI-Q-TOF-MS spectrum of compound **3** 13
Fig. S22 UV spectrum of compound **3**...13
Fig. S23 IR spectrum of compound **3**...13
Fig. S24 ^{1}H-NMR spectrum of compound **3**..14
Fig. S25 ^{13}C-NMR spectrum of compound **3**...14
Fig. S26 DEPT 135 spectrum of compound **3**.......................... 15
Fig. S27 ^{1}H-^{1}H COSY spectrum of compound **3**...15
Fig. S28 HSQC spectrum of compound **3**..16
Fig. S29 HMBC spectrum of compound **3**...16
Fig. S30 ROESY spectrum of compound **3**..17
Fig. S31 HR ESI-Q-TOF-MS spectrum of compound **4**...17
Fig. S32 UV spectrum of compound **4**...18
Fig. S33 IR spectrum of compound **4**...18

Fig. S34 ^{1}H-NMR spectrum of compound **4**..18
Fig. S35 ^{13}C-NMR spectrum of compound **4**...19
Fig. S36 DEPT 135 spectrum of compound **4**............................. 19
Fig. S37 ^{1}H-^{1}H COSY spectrum of compound **4**...20
Fig. S38 HSQC spectrum of compound **4**..20
Fig. S39 HMBC spectrum of compound **4**...21
Fig. S40 **ROESY** spectrum of compound **4**..21
Fig. S41 HR ESI-Q-TOF-MS spectrum of compound **5**............................. 22
Fig. S42 UV spectrum of compound **5**...22
Fig. S43 IR spectrum of compound **5**...22
Fig. S44 ^{1}H-NMR spectrum of compound **5**...23
Fig. S45 ^{13}C-NMR spectrum of compound **5**..23
Fig. S46 DEPT 135 spectrum of compound **5**............................. 24
Fig. S47 ^{1}H-^{1}H COSY spectrum of compound **5**..24
Fig. S48 HSQC spectrum of compound **5**..25
Fig. S49 HMBC spectrum of compound **5**... 25
Fig. S50 **ROESY** spectrum of compound **5**...26
Fig. S51 Cultural and microscopic morphology of strain WHU0154.......................27

Elemental Composition Report **Page 1**

Single Mass Analysis
Tolerance = 5.0 PPM / DBE: min = -1.5, max = 50.0
Element prediction: Off
Number of isotope peaks used for i-FIT = 3

Monoisotopic Mass, Even Electron Ions
849 formula(e) evaluated with 2 results within limits (up to 50 closest results for each mass)
Elements Used:
C: 0-50 H: 0-100 N: 0-10 O: 0-100
0154-78-67-5-5
20191216009 172 (1.388)

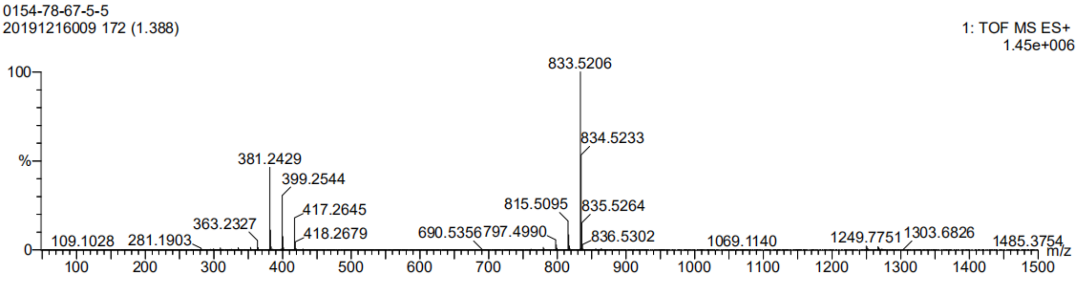

Minimum:				-1.5				
Maximum:		5.0	5.0	50.0				

Mass	Calc. Mass	mDa	PPM	DBE	i-FIT	Norm	Conf(%)	Formula
417.2645	417.2641	0.4	1.0	7.5	384.9	0.219	80.36	C25 H37 O5
	417.2654	-0.9	-2.2	12.5	386.4	1.628	19.64	C26 H33 N4 O

Fig. S1 HR ESI-Q-TOF-MS spectrum of compound **1**

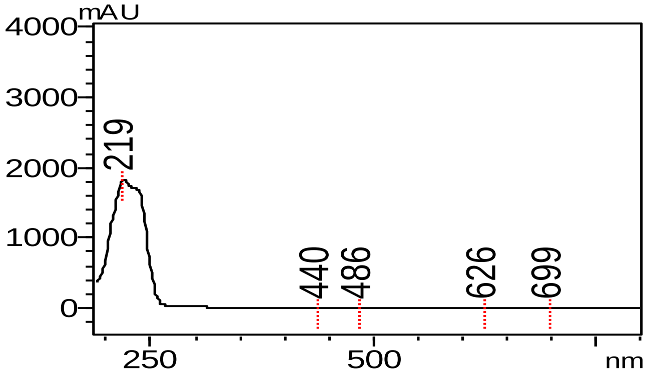

Fig. S2 UV spectrum of compound **1**

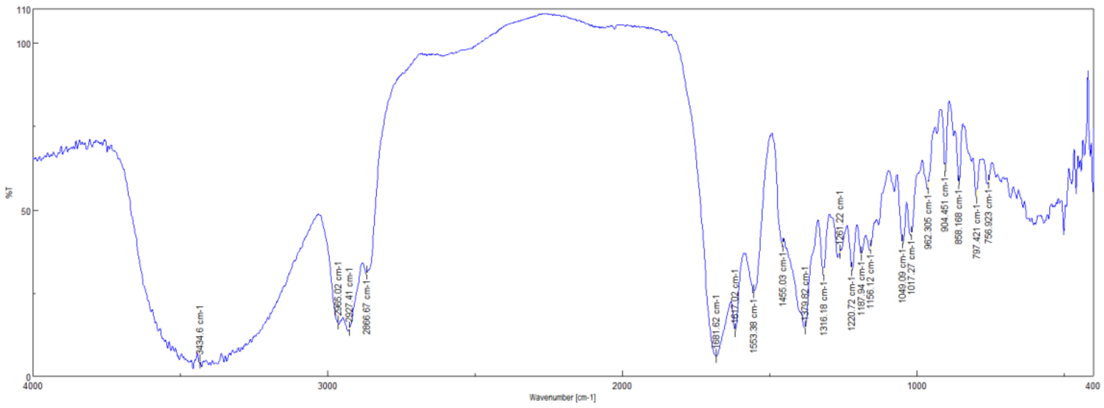

Fig. S3 IR spectrum of compound **1**

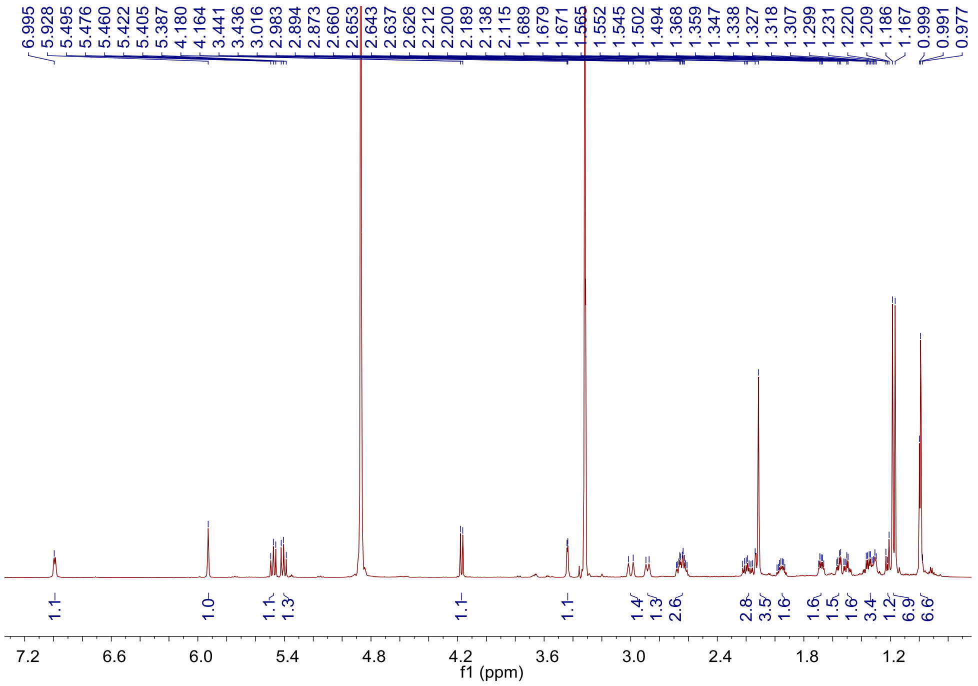

Fig. S4 ¹H-NMR spectrum of compound **1** (in CD_3OD)

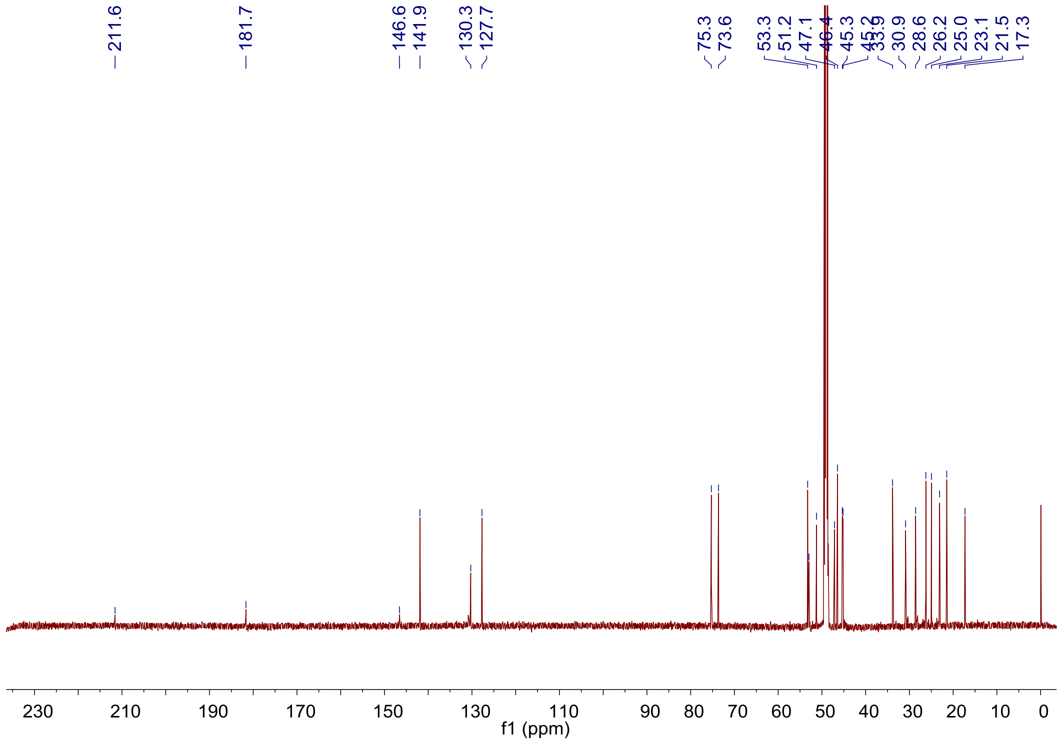

Fig. S5 ¹³C-NMR spectrum of compound **1** (in CD_3OD)

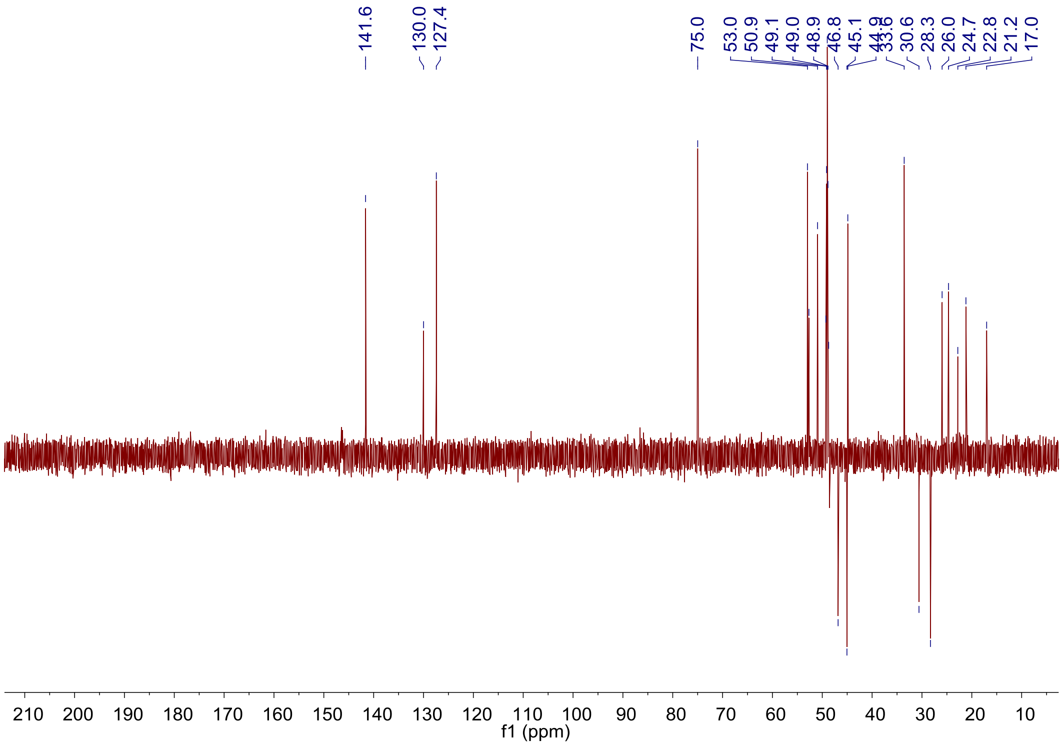

Fig. S6 DEPT135 spectrum of compound **1** (in CD_3OD)

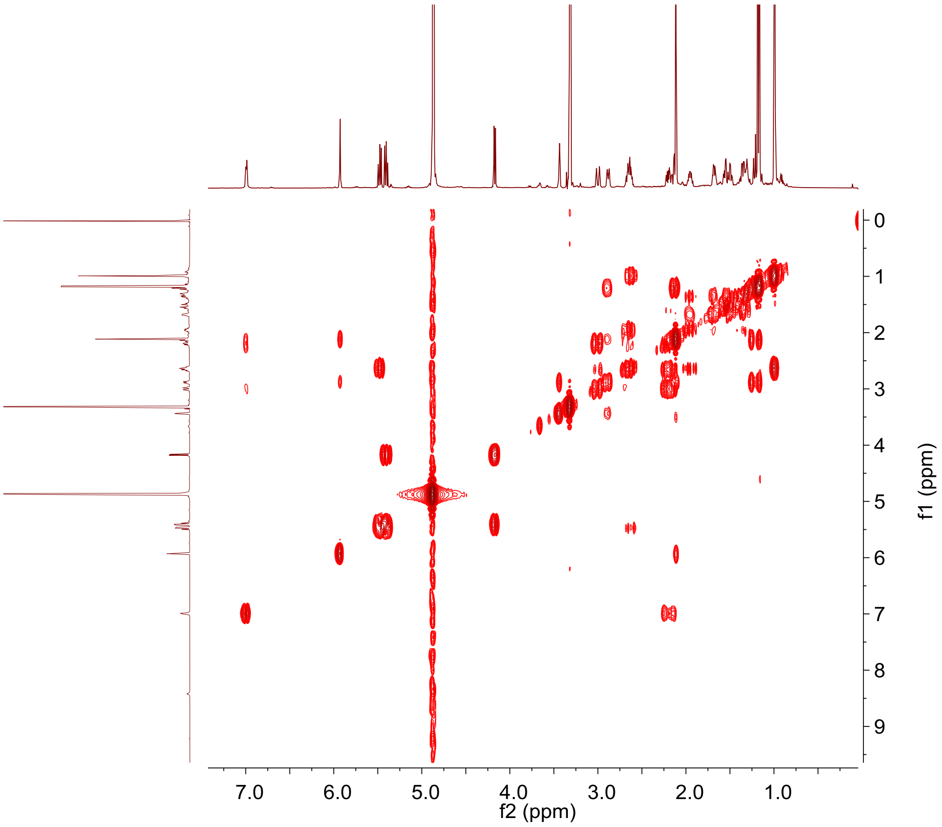

Fig. S7 ^{1}H-^{1}H COSY spectrum of compound **1** (in CD_3OD)

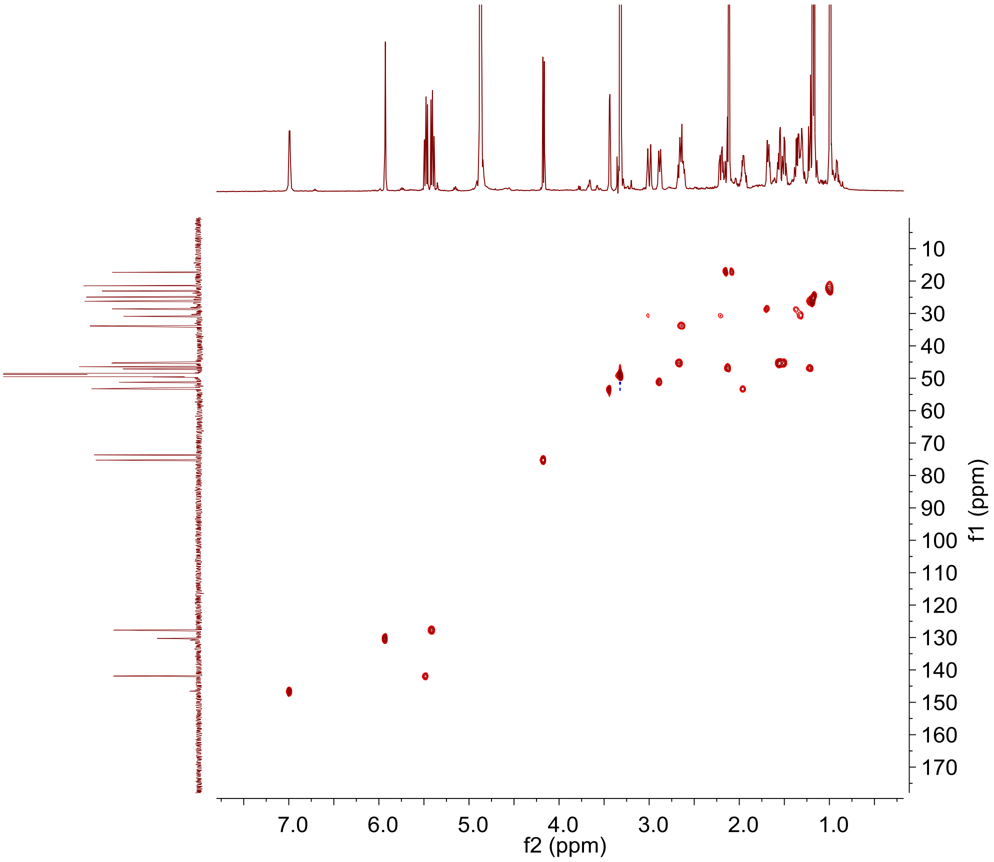

Fig. S8 HSQC spectrum of compound **1** (in CD_3OD)

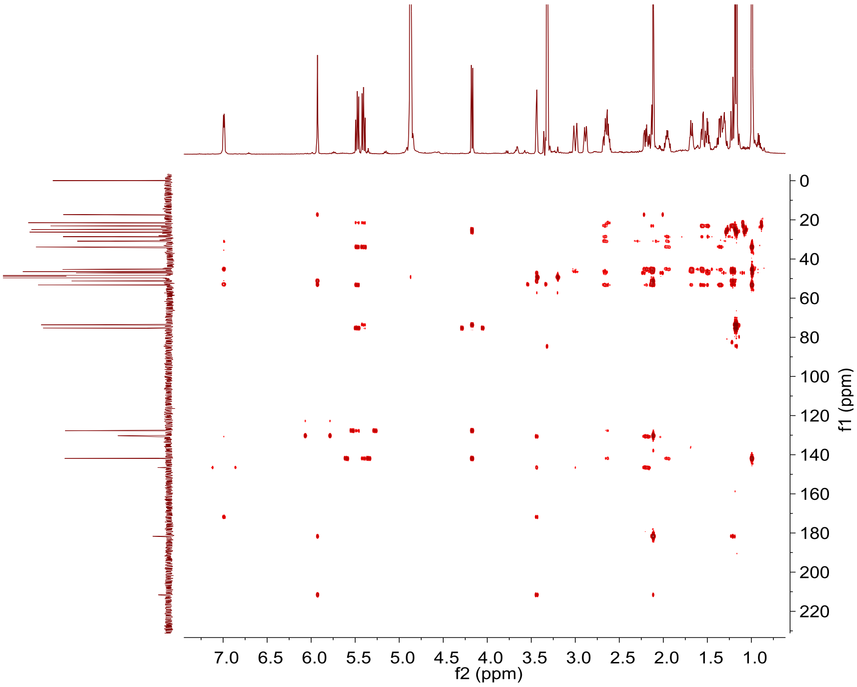

Fig. S9 HMBC spectrum of compound **1** (in CD3OD).

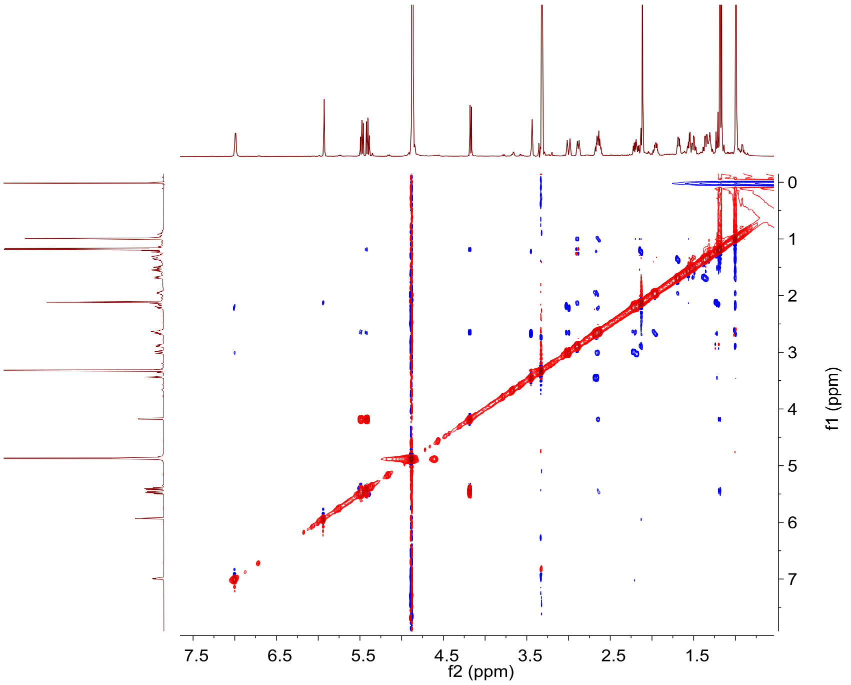

Fig. S10 ROESY spectrum of compound **1** (in CD3OD).

Elemental Composition Report **Page 1**

Single Mass Analysis
Tolerance = 5.0 PPM / DBE: min = -1.5, max = 50.0
Element prediction: Off
Number of isotope peaks used for i-FIT = 3

Monoisotopic Mass, Even Electron Ions
728 formula(e) evaluated with 1 results within limits (up to 50 closest results for each mass)
Elements Used:
C: 0-50 H: 0-100 N: 0-10 O: 0-100
0154-6-9-2-7
20191202054 291 (2.345)

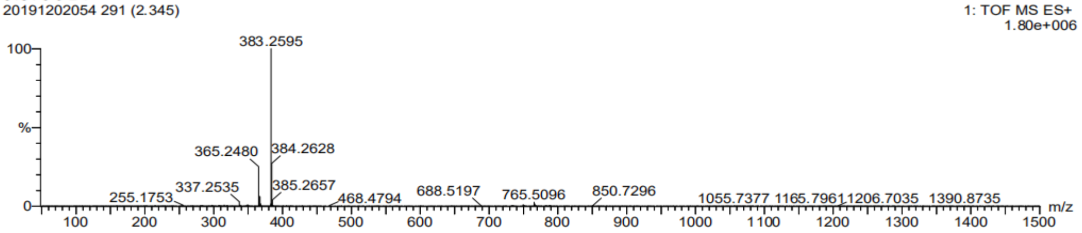

Minimum:				-1.5				
Maximum:		5.0	5.0	50.0				
Mass	Calc. Mass	mDa	PPM	DBE	i-FIT	Norm	Conf(%)	Formula
383.2595	383.2586	0.9	2.3	8.5	752.1	n/a	n/a	C25 H35 O3

Fig. S11 HR ESI-Q-TOF-MS spectrum of compound **2**

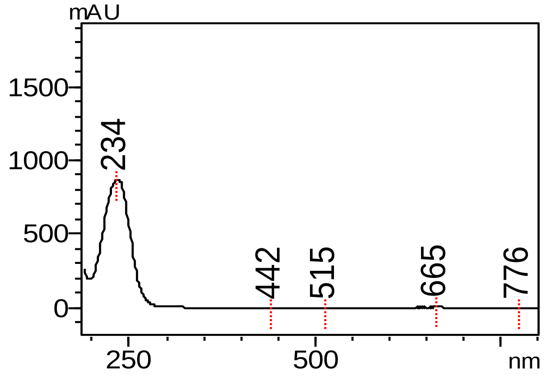

Fig. S12 UV spectrum of compound **2**

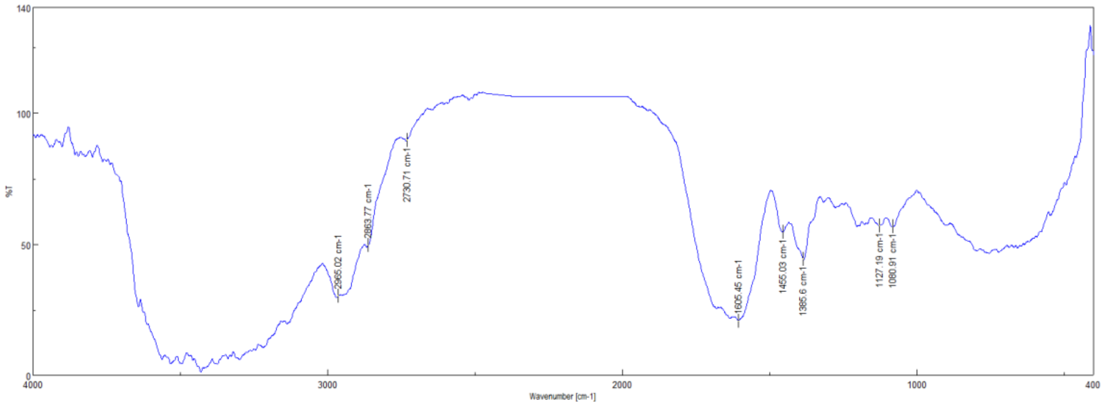

Fig. S13 IR spectrum of compound **2**

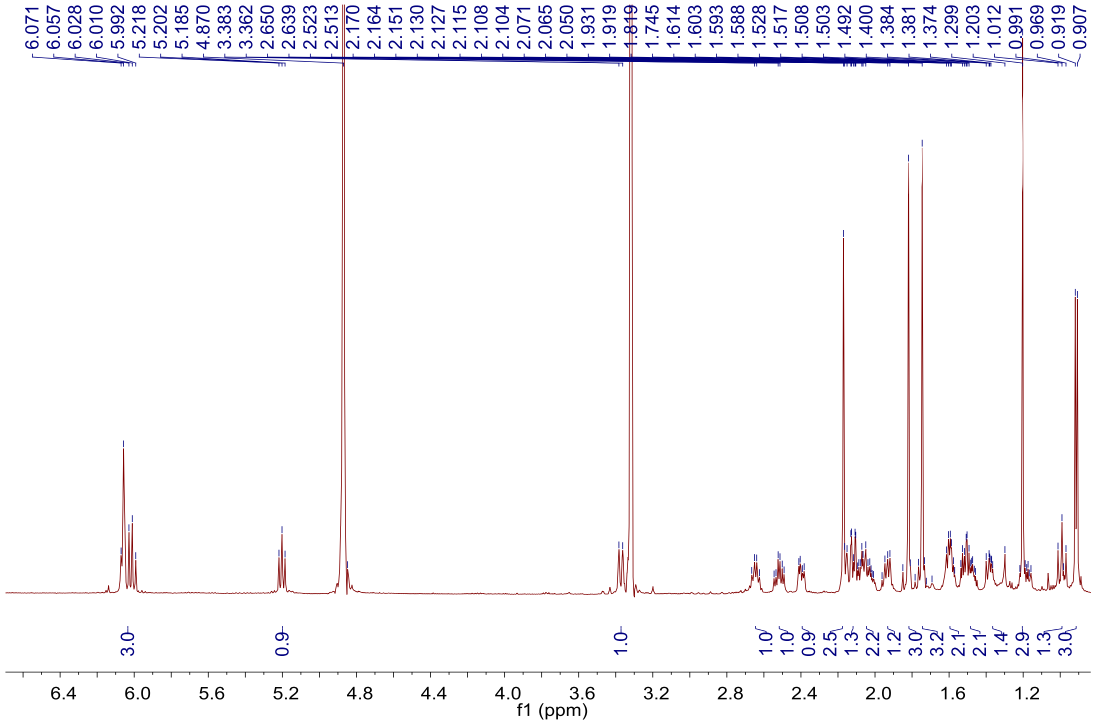

Fig. S14 ^{1}H-NMR spectrum of compound **2** (in CD_3OD)

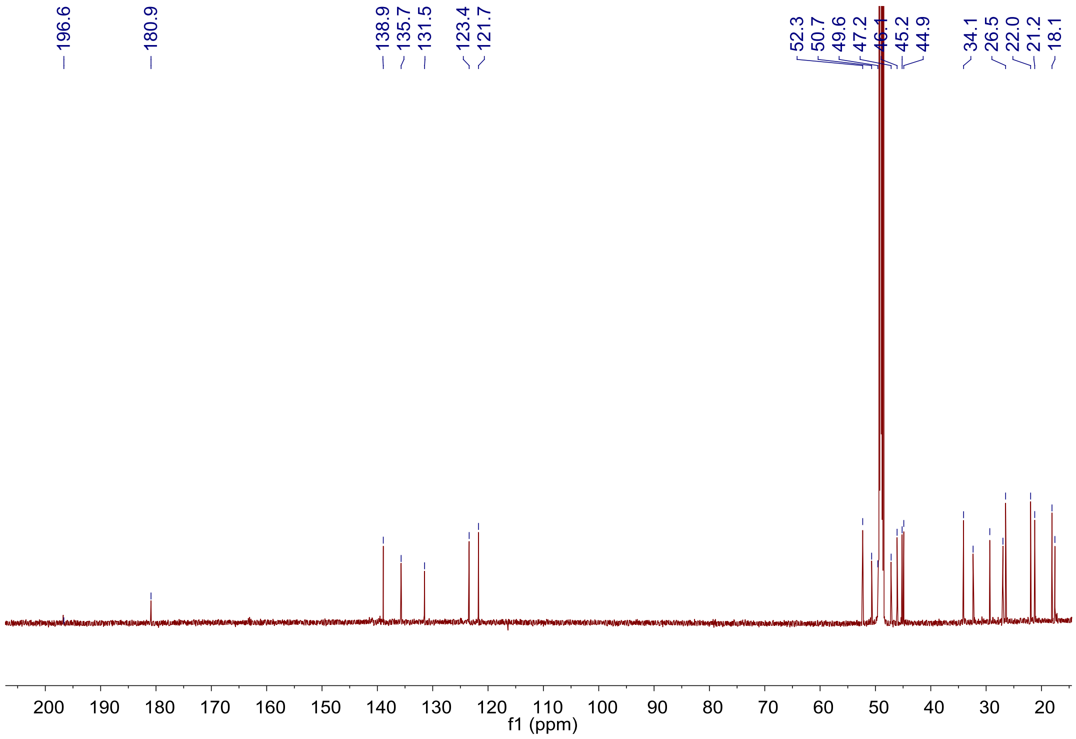

Fig. S15 ^{13}C-NMR spectrum of compound **2** (in CD_3OD)

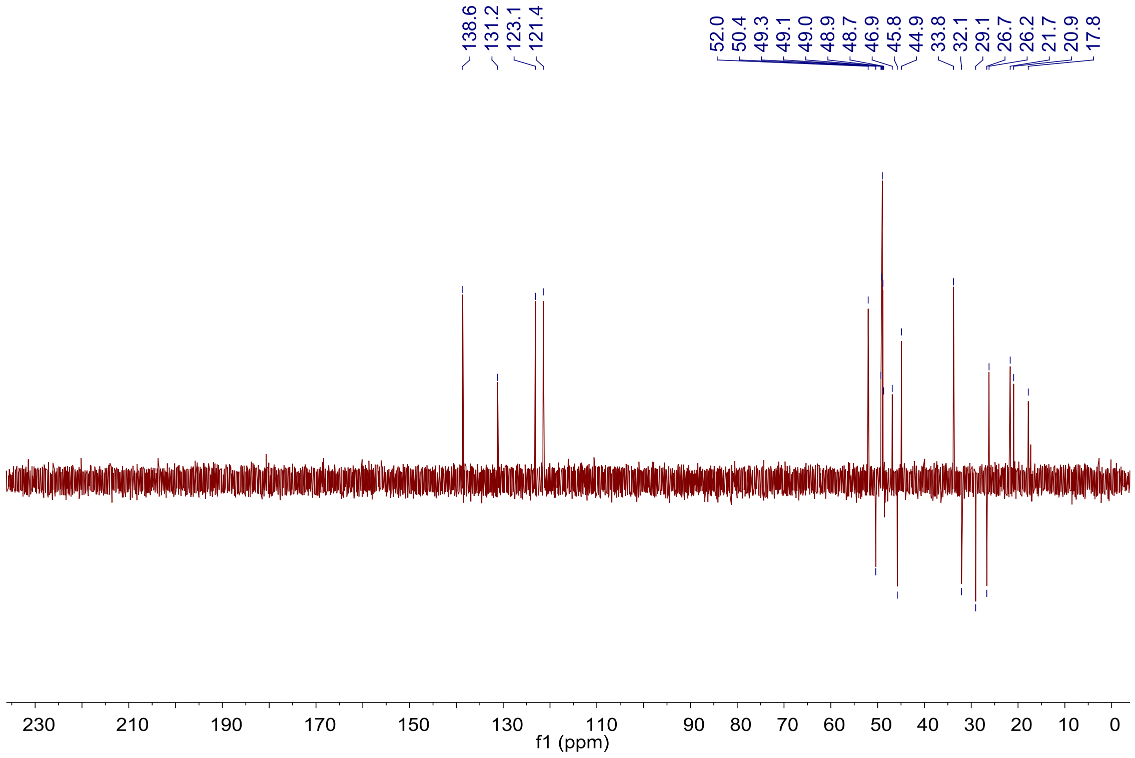

Fig. S16 DEPT135 spectrum of compound **2** (in CD_3OD)

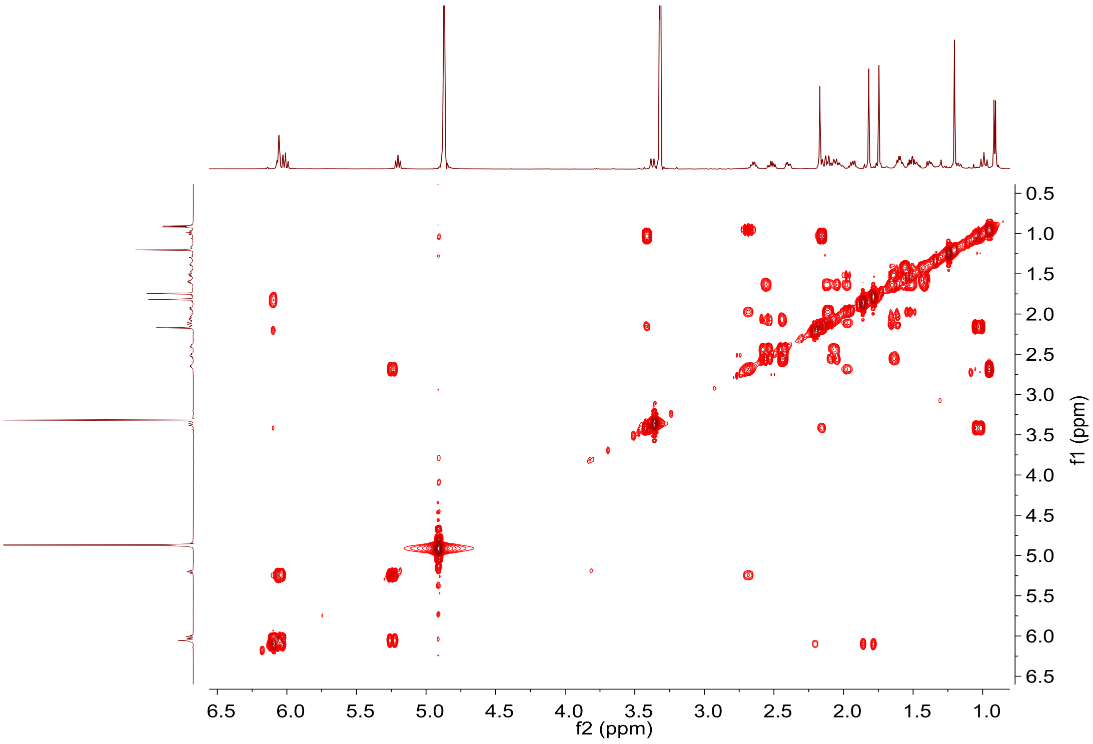

Fig. S17 ^{1}H-^{1}H COSY spectrum of compound **2** (in CD_3OD)

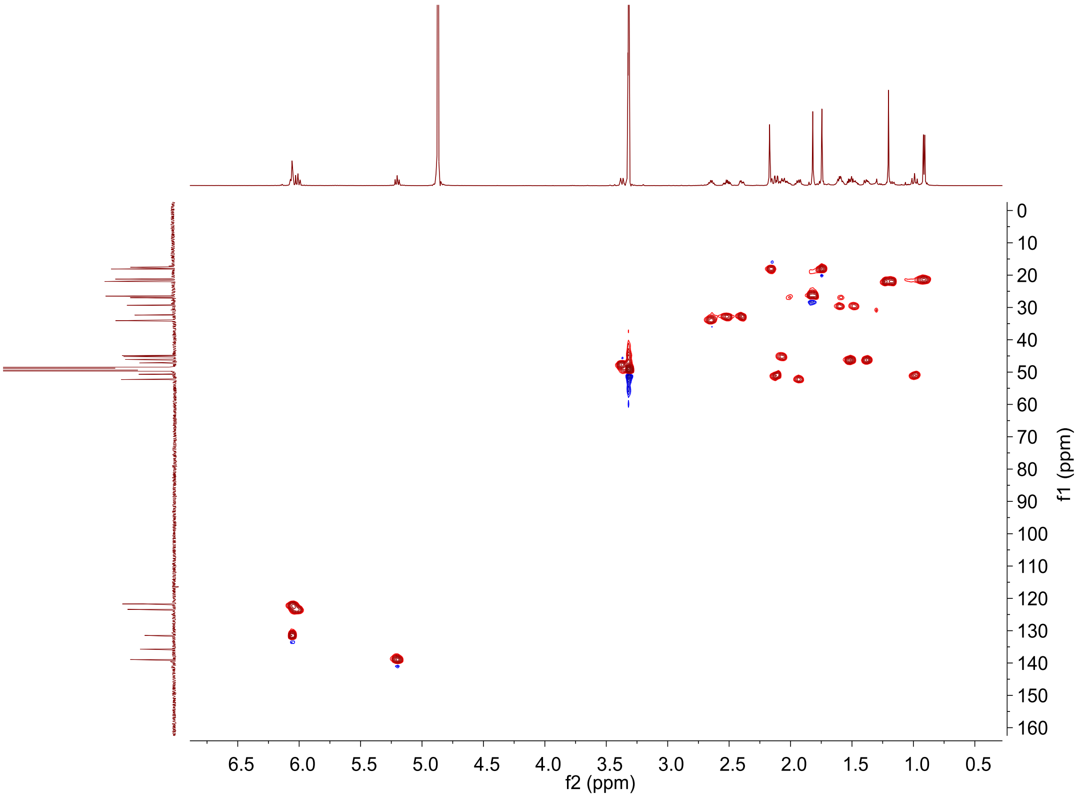

Fig. S18 HSQC spectrum of compound **2** (in CD_3OD)

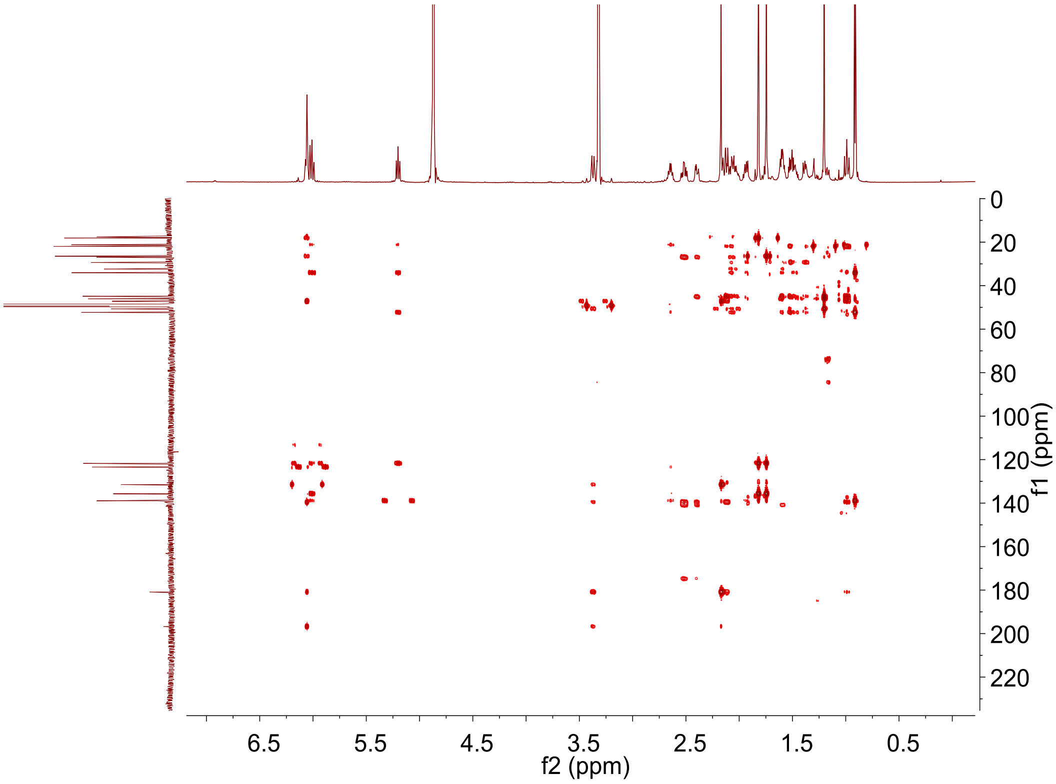

Fig. S19 HMBC spectrum of compound **2** (in CD3OD)

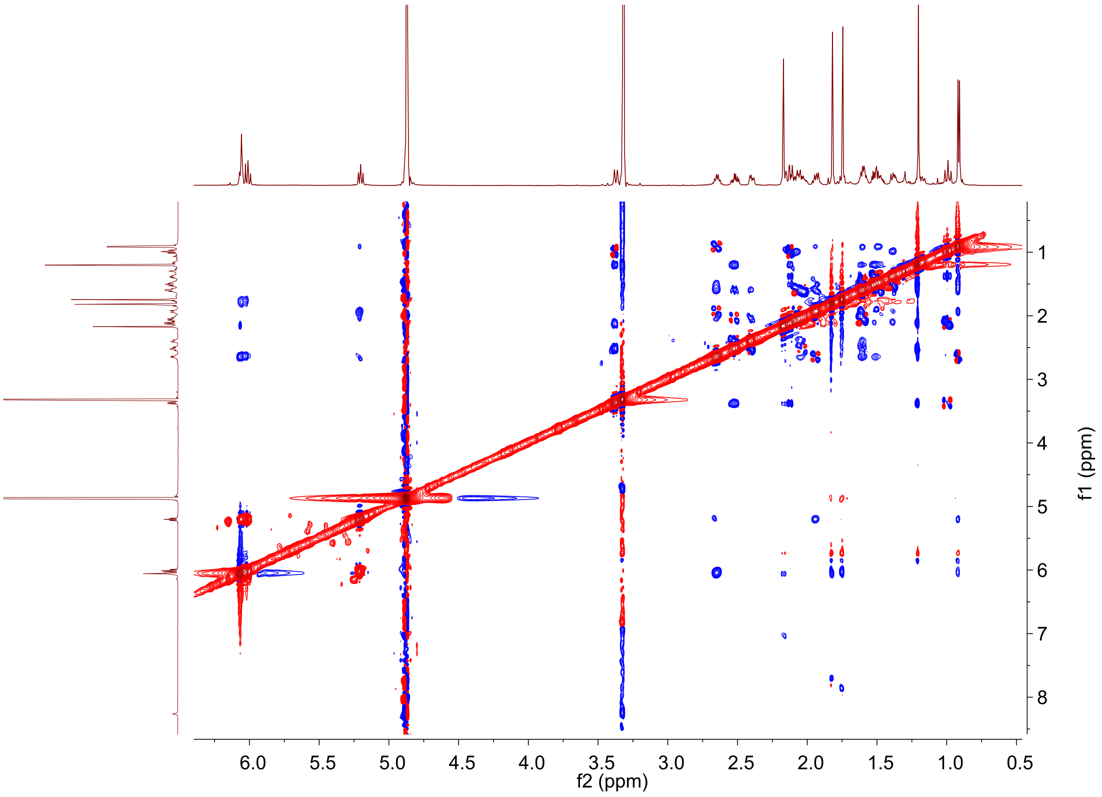

Fig. S20 ROESY spectrum of compound **2** (in CD3OD).

Elemental Composition Report Page 1

Single Mass Analysis
Tolerance = 5.0 mDa / DBE: min = -1.5, max = 50.0
Element prediction: Off
Number of isotope peaks used for i-FIT = 3

Monoisotopic Mass, Even Electron Ions
89 formula(e) evaluated with 1 results within limits (up to 50 best isotopic matches for each mass)
Elements Used:
C: 0-500 H: 0-1000 O: 0-200
0154-78-8-2
20191104042 255 (2.053) 1: TOF MS ES+ 9.83e+005

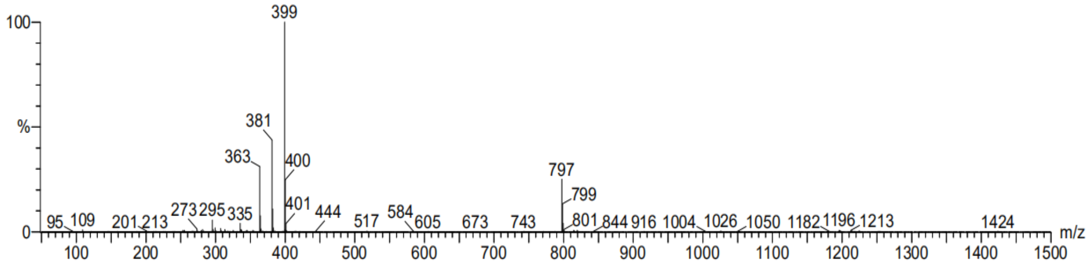

Minimum:				-1.5				
Maximum:		5.0	10.0	50.0				
Mass	Calc. Mass	mDa	PPM	DBE	i-FIT	Norm	Conf(%)	Formula
399.2537	399.2535	0.2	0.5	8.5	599.0	n/a	n/a	C25 H35 O4

Fig. S21 HR ESI-Q-TOF-MS spectrum of compound **3**

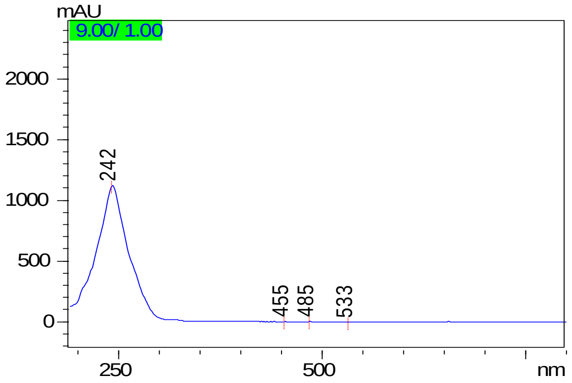

Fig. S22 UV spectrum of compound **3**

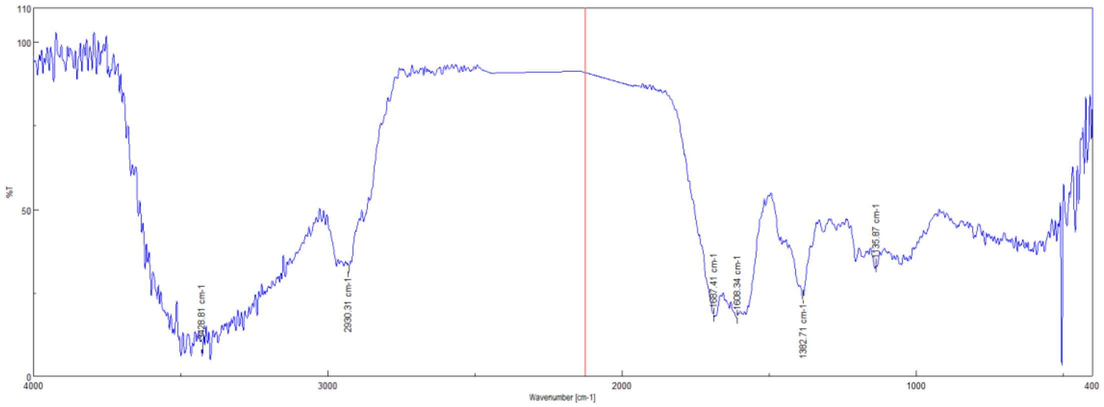

Fig. S23 IR spectrum of compound **3**

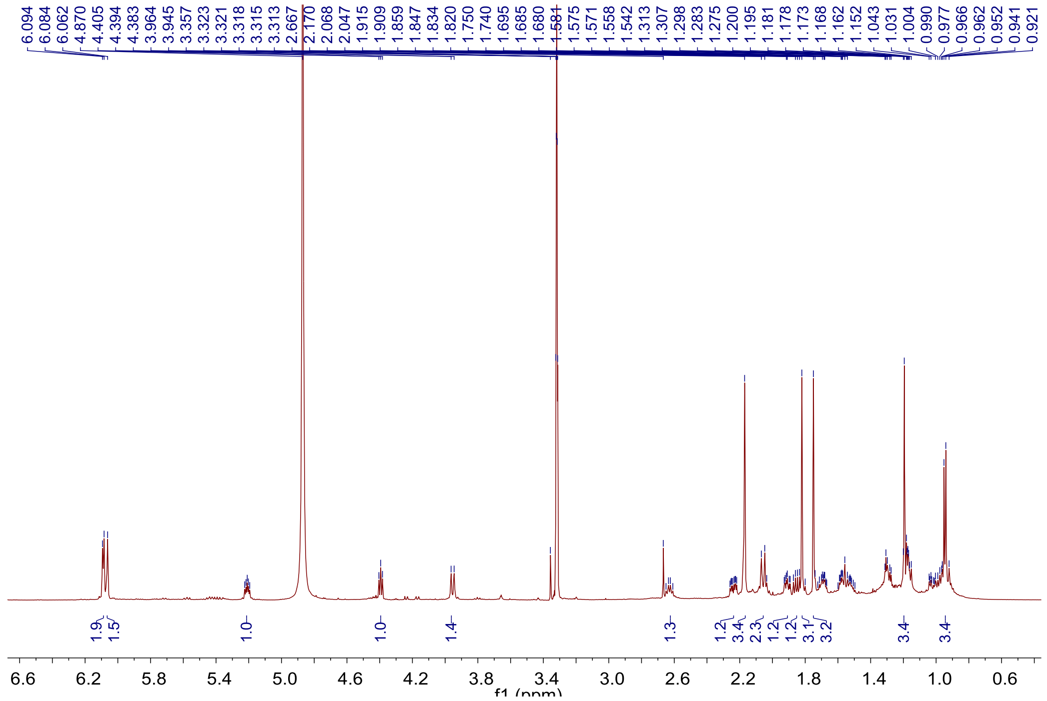

Fig. S24 ^{1}H-NMR spectrum of compound **3** (in CD_3OD)

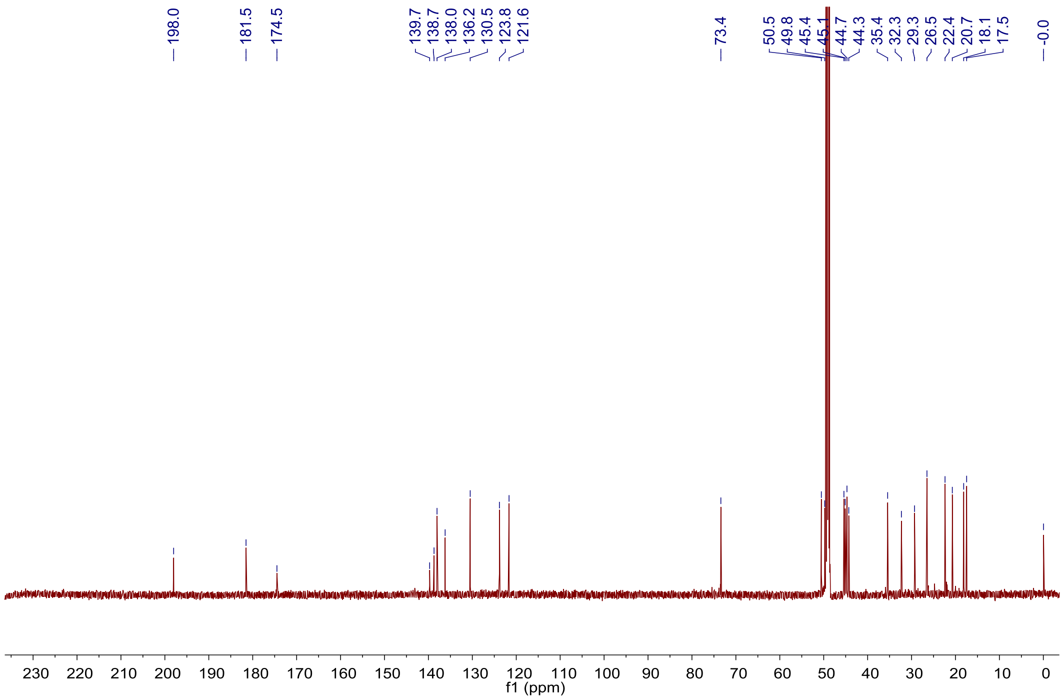

Fig. S25 ^{13}C-NMR spectrum of compound **3** (in CD_3OD)

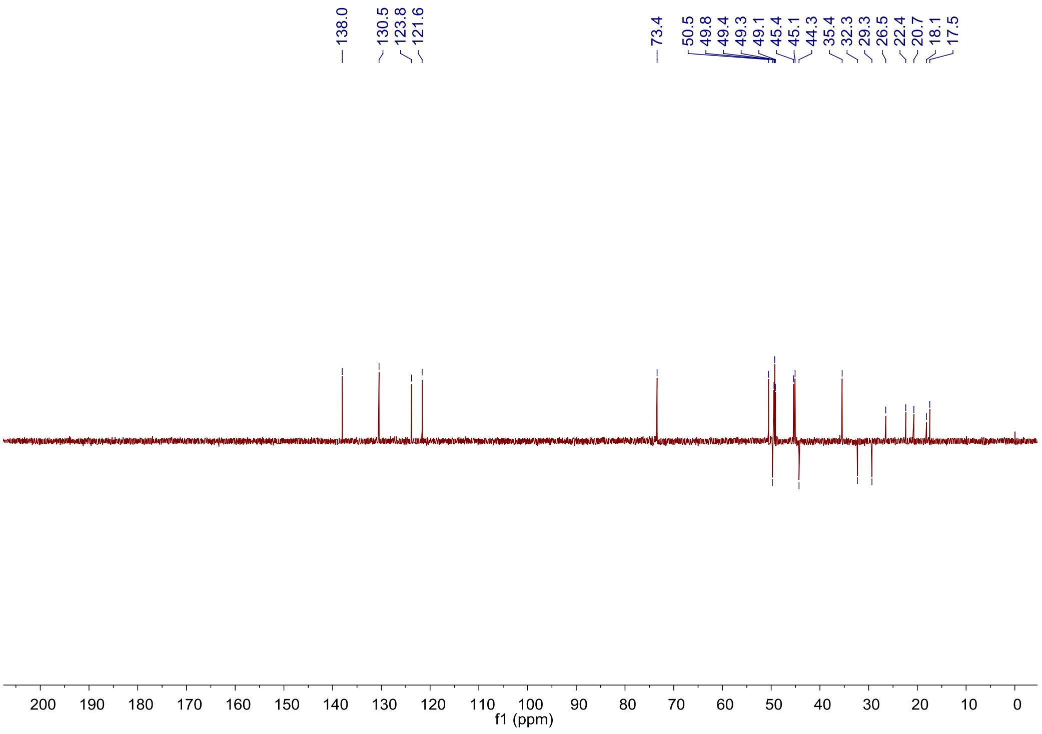

Fig. S26 DEPT135 spectrum of compound **3** (in CD_3OD)

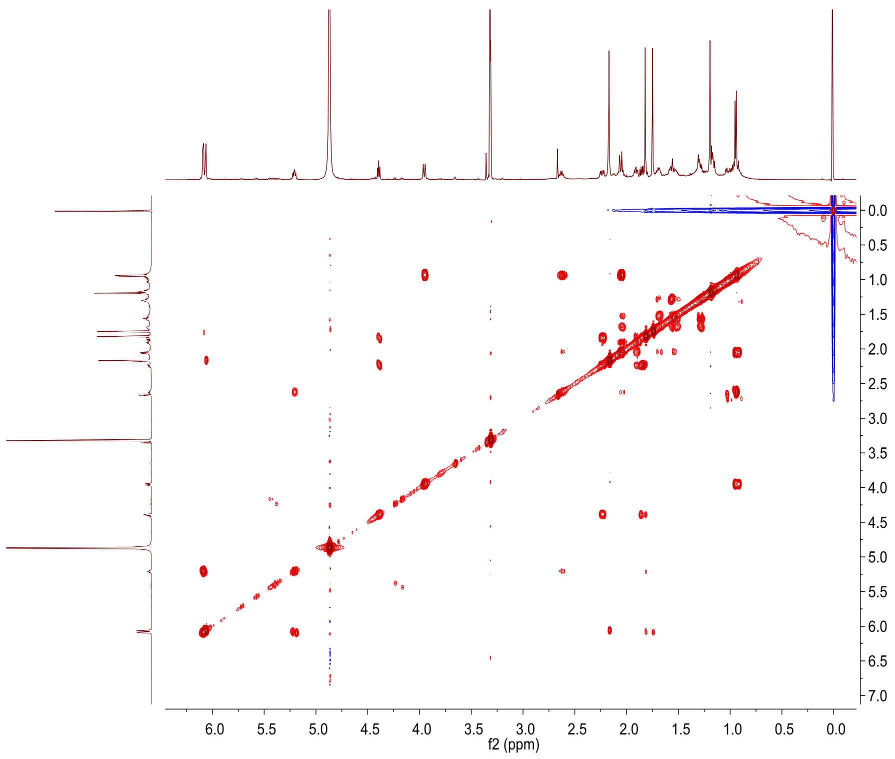

Fig. S27 ^1H-^1H COSY spectrum of compound **3** (in CD_3OD)

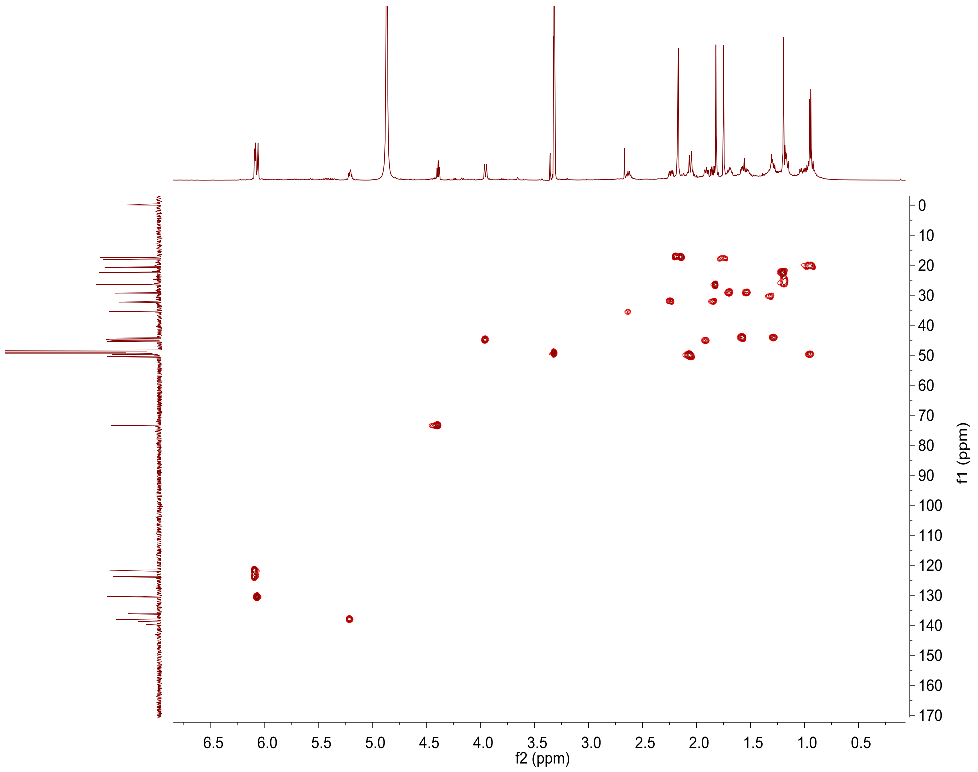

Fig. S28 HSQC spectrum of compound **3** (in CD_3OD)

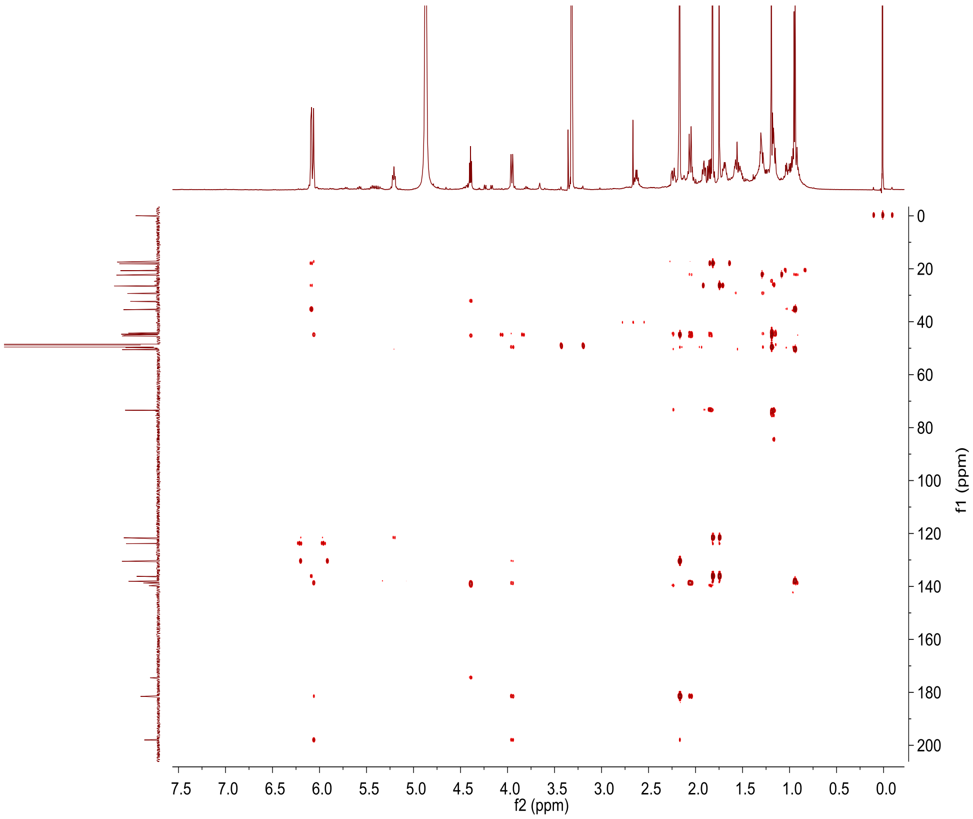

Fig. S29 HMBC spectrum of compound **3** (in CD3OD).

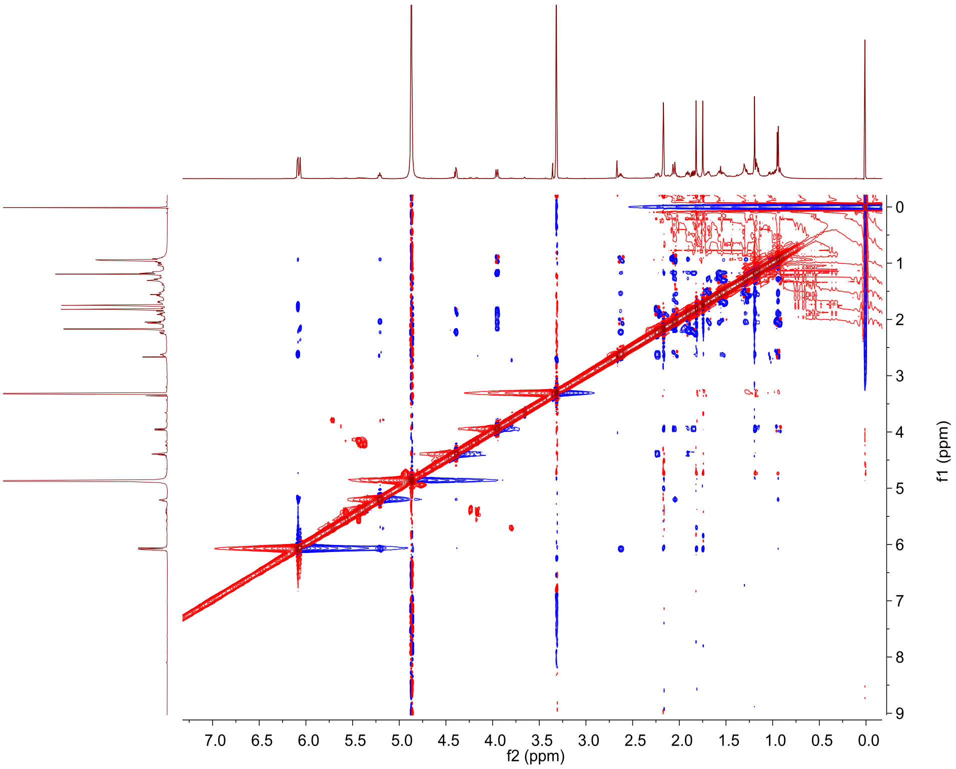

Fig. S30 ROESY spectrum of compound **3** (in CD3OD).

Elemental Composition Report **Page 1**

Single Mass Analysis
Tolerance = 5.0 PPM / DBE: min = -1.5, max = 50.0
Element prediction: Off
Number of isotope peaks used for i-FIT = 3

Monoisotopic Mass, Even Electron Ions
2369 formula(e) evaluated with 7 results within limits (up to 50 best isotopic matches for each mass)
Elements Used:
C: 0-50 H: 0-100 N: 0-10 O: 0-100
0154-78-8-4
20191104041 255 (2.053) 1: TOF MS ES+
5.14e+005

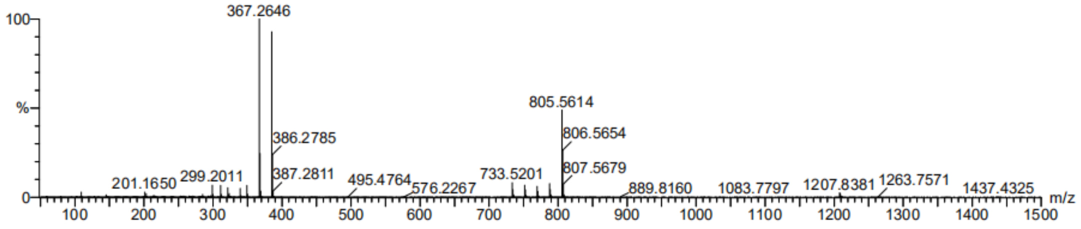

Mass	Calc. Mass	mDa	PPM	DBE	i-FIT	Norm	Conf(%)	Formula
Minimum:				-1.5				
Maximum:		5.0	5.0	50.0				
805.5614	805.5605	0.9	1.1	18.5	417.0	1.874	15.35	C47 H69 N10 O2
	805.5592	2.2	2.7	13.5	415.3	0.199	81.97	C46 H73 N6 O6
	805.5618	-0.4	-0.5	12.5	418.9	3.819	2.19	C50 H77 O8
	805.5578	3.6	4.5	8.5	420.5	5.399	0.45	C45 H77 N2 O10
	805.5650	-3.6	-4.5	4.5	424.2	9.084	0.01	C39 H77 N6 O11
	805.5610	0.4	0.5	0.5	424.7	9.620	0.01	C34 H77 N8 O13
	805.5637	-2.3	-2.9	-0.5	423.8	8.753	0.02	C38 H81 N2 O15

Fig. S31 HR ESI-Q-TOF-MS spectrum of compound **4**

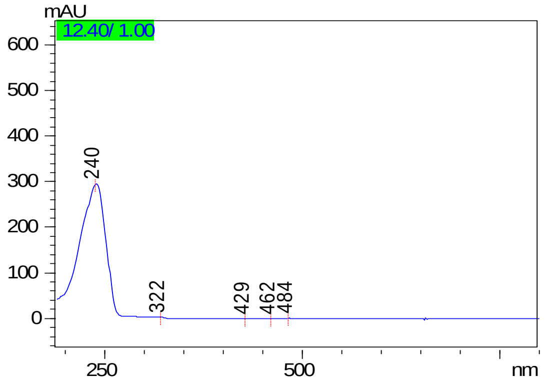

Fig. S32 UV spectrum of compound **4**

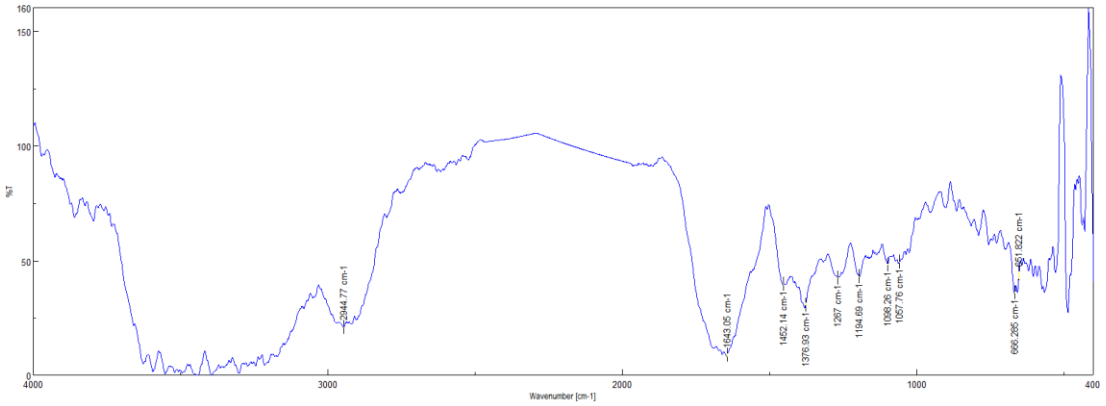

Fig. S33 IR spectrum of compound **4**

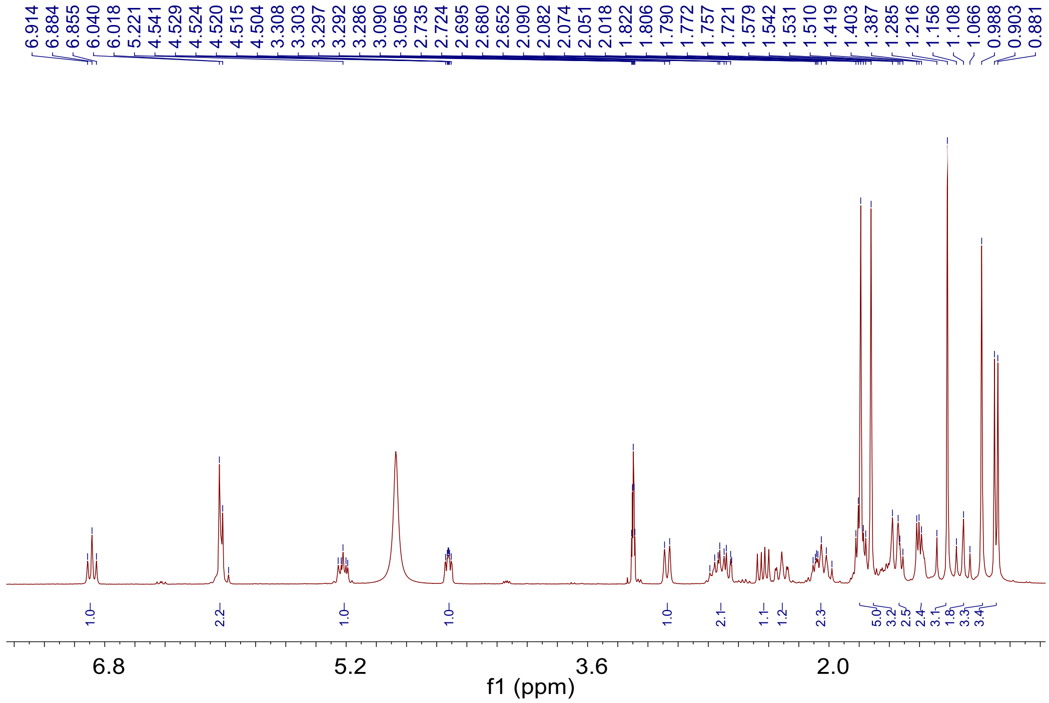

Fig. S34 ^{1}H-NMR spectrum of compound **4** (in CD_3OD)

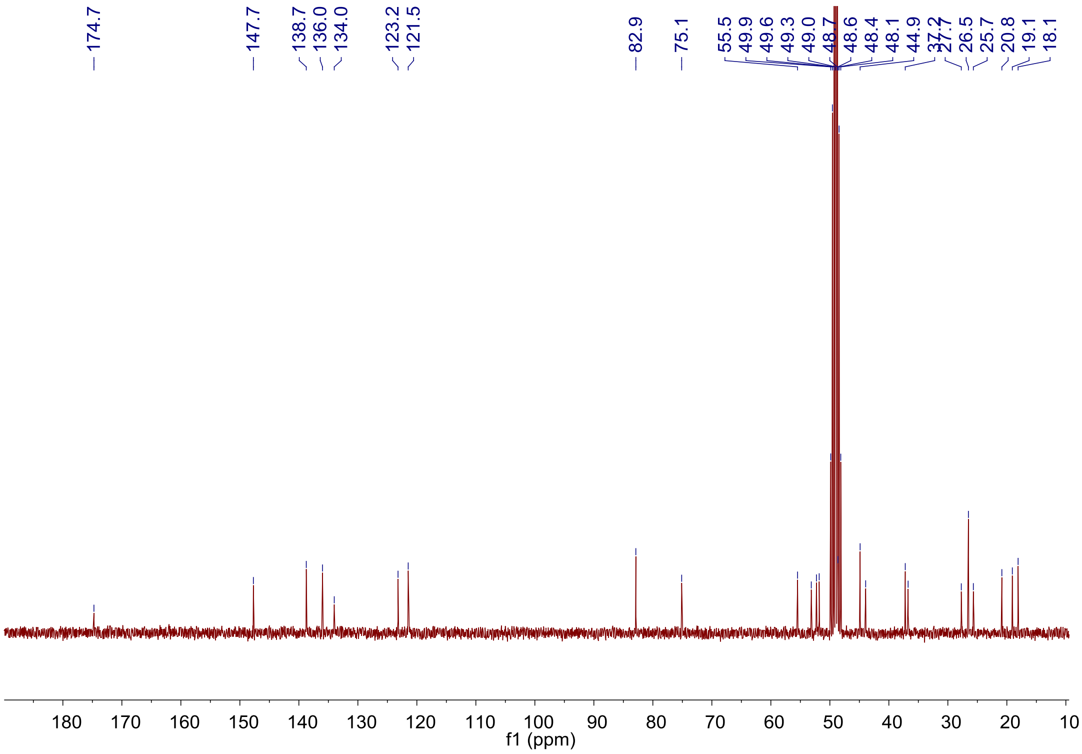

Fig. S35 ^{13}C-NMR spectrum of compound **4** (in CD_3OD)

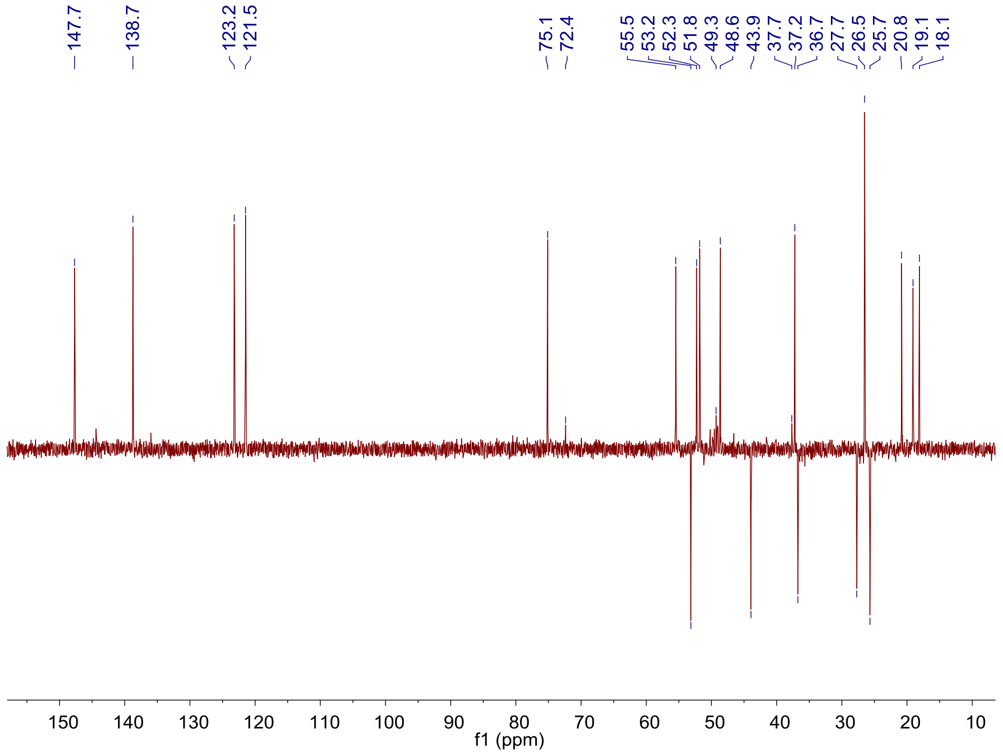

Fig. S36 DEPT135 spectrum of compound **4** (in CD_3OD)

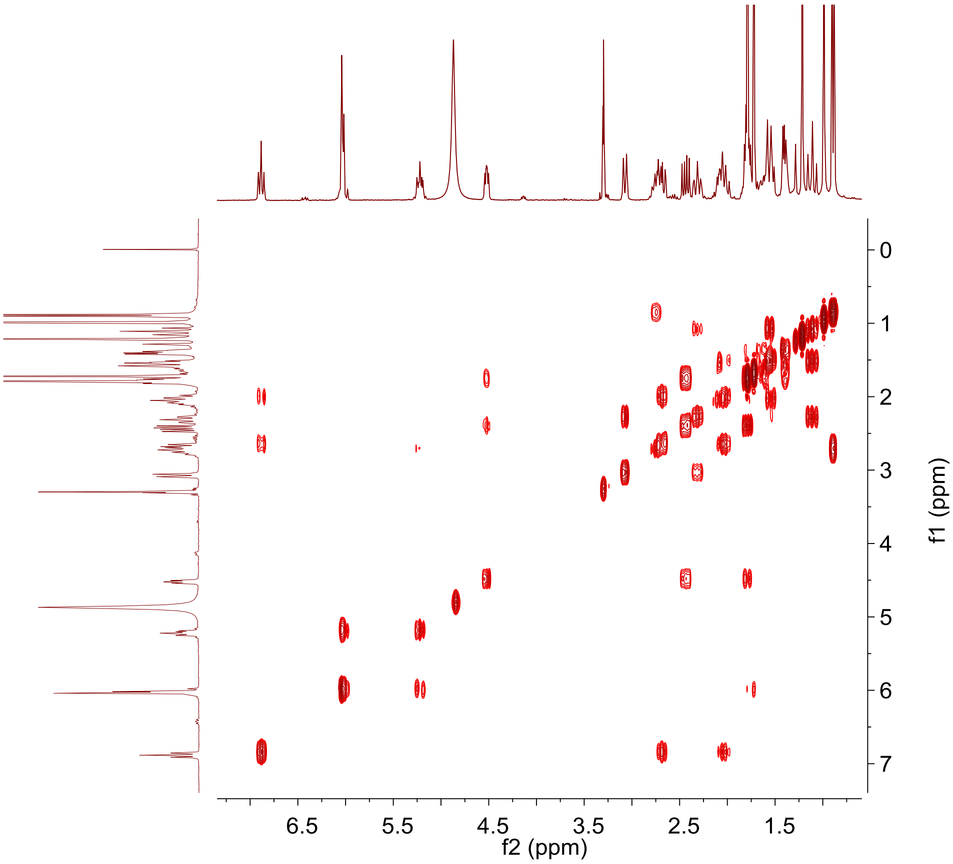

Fig. S37 ^{1}H-^{1}H COSY spectrum of compound **4** (in CD_3OD)

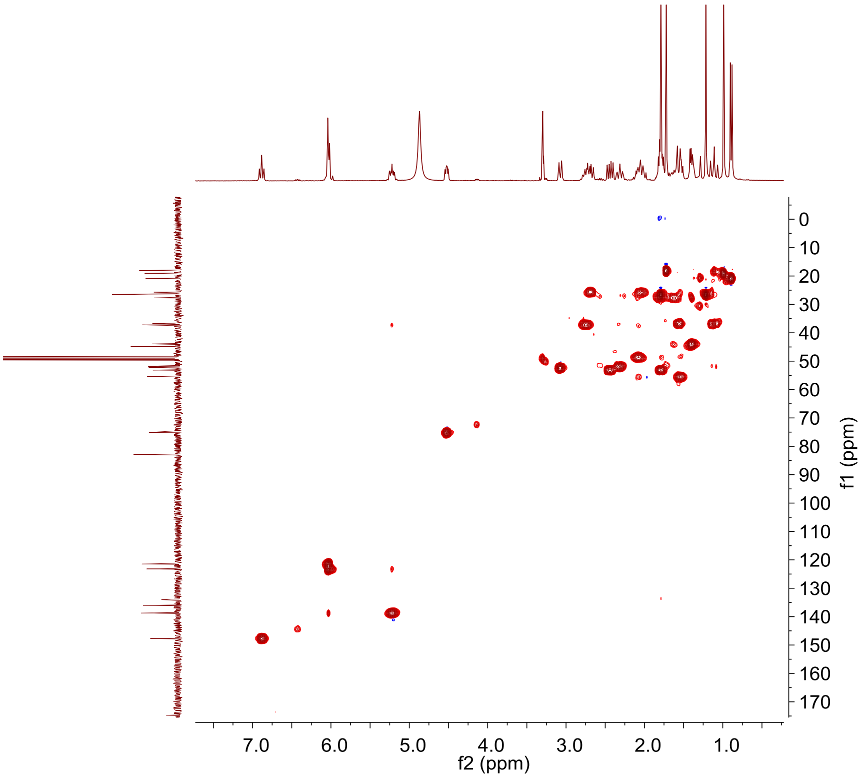

Fig. S38 HSQC spectrum of compound **4** (in CD_3OD)

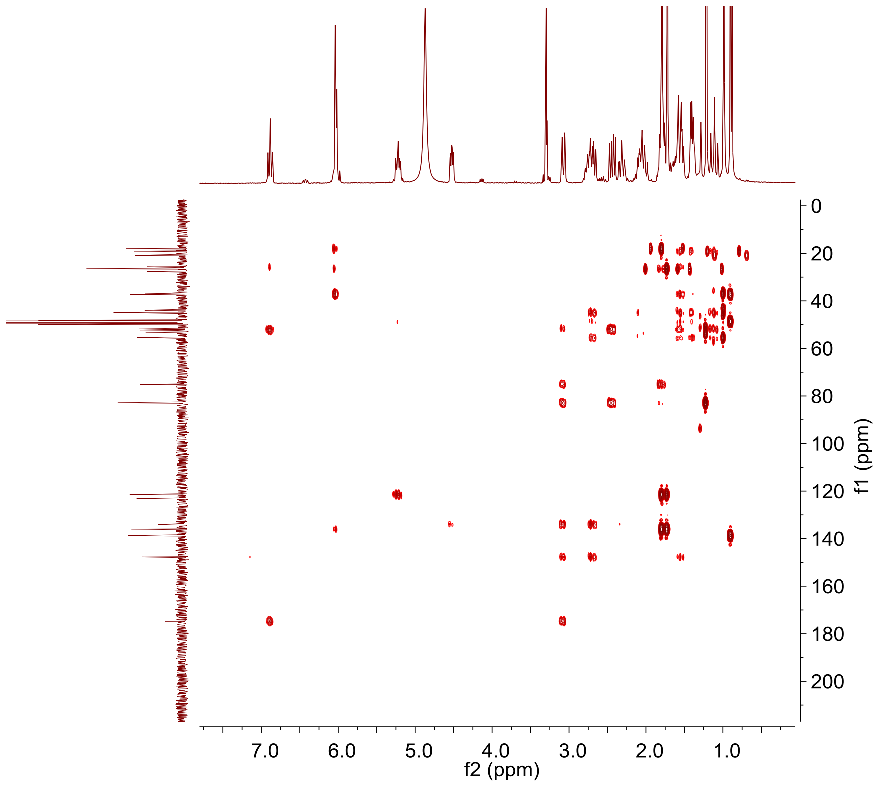

Fig. S39 HMBC spectrum of compound **4** (in CD3OD).

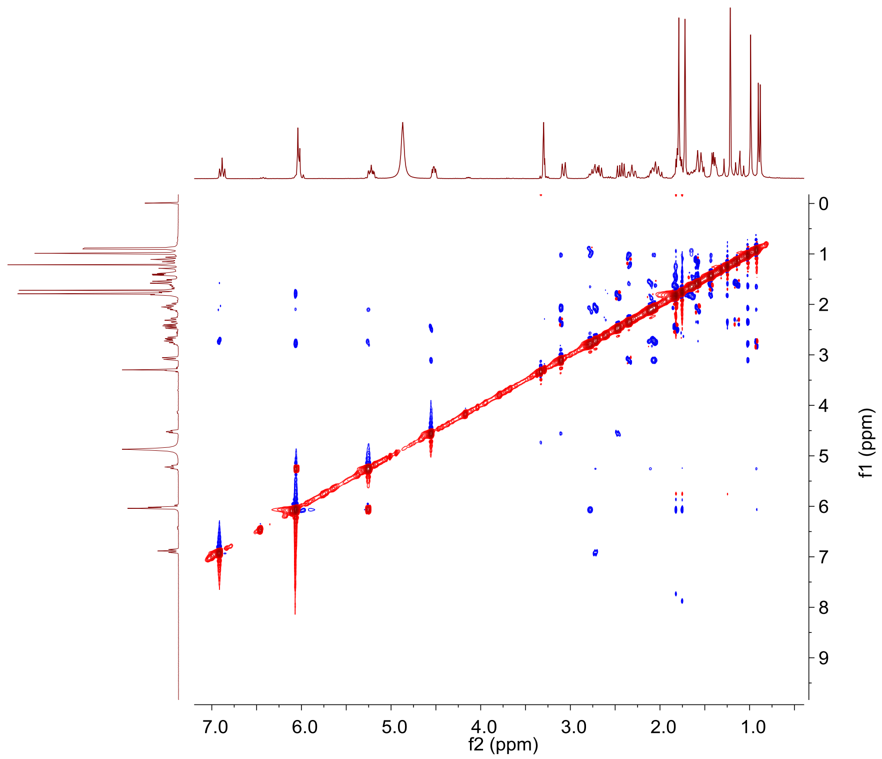

Fig. S40 ROESY spectrum of compound **4** (in CD3OD).

Elemental Composition Report Page 1

Single Mass Analysis
Tolerance = 5.0 PPM / DBE: min = -1.5, max = 50.0
Element prediction: Off
Number of isotope peaks used for i-FIT = 3

Monoisotopic Mass, Even Electron Ions
4513 formula(e) evaluated with 11 results within limits (up to 50 closest results for each mass)
Elements Used:
C: 0-50 H: 0-200 N: 0-20 O: 0-100
0154-78-8-5
20191104039 256 (2.061) 1: TOF MS ES+ 1.29e+005

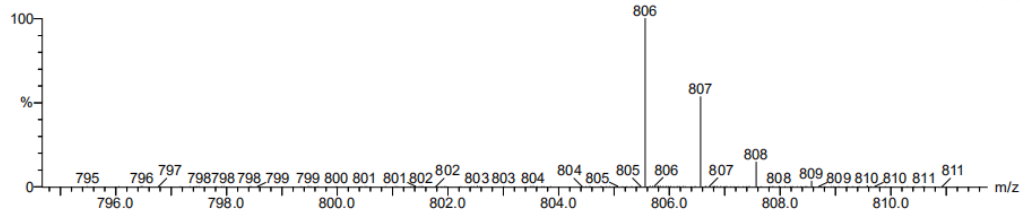

Minimum:				-1.5				
Maximum:		5.0	5.0	50.0				

Mass	Calc. Mass	mDa	PPM	DBE	i-FIT	Norm	Conf(%)	Formula
805.5621	805.5623	-0.2	-0.2	5.5	343.2	7.886	0.04	C35 H73 N12 O9
	805.5618	0.3	0.4	12.5	340.1	4.842	0.79	C50 H77 O8
	805.5610	1.1	1.4	0.5	344.0	8.765	0.02	C34 H77 N8 O13
	805.5637	-1.6	-2.0	10.5	343.0	7.749	0.04	C36 H69 N16 O5
	805.5637	-1.6	-2.0	-0.5	344.4	9.107	0.01	C38 H81 N2 O15
	805.5605	1.6	2.0	18.5	337.2	1.943	14.33	C47 H69 N10 O2
	805.5597	2.4	3.0	6.5	345.9	10.624	0.00	C31 H69 N18 O7
	805.5650	-2.9	-3.6	15.5	343.4	8.100	0.03	C37 H65 N20 O
	805.5650	-2.9	-3.6	4.5	344.5	9.200	0.01	C39 H77 N6 O11

Fig. S41 HR ESI-Q-TOF-MS spectrum of compound **5**

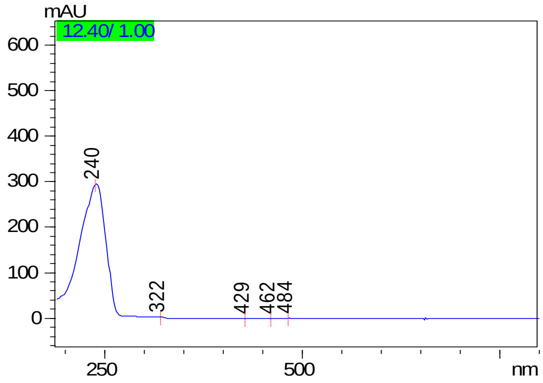

Fig. S42 UV spectrum of compound **5**

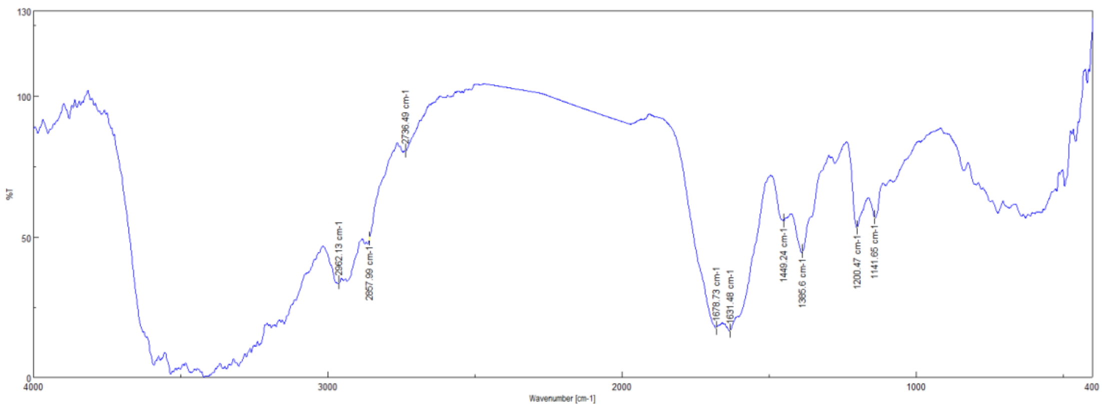

Fig. S43 IR spectrum of compound **5**

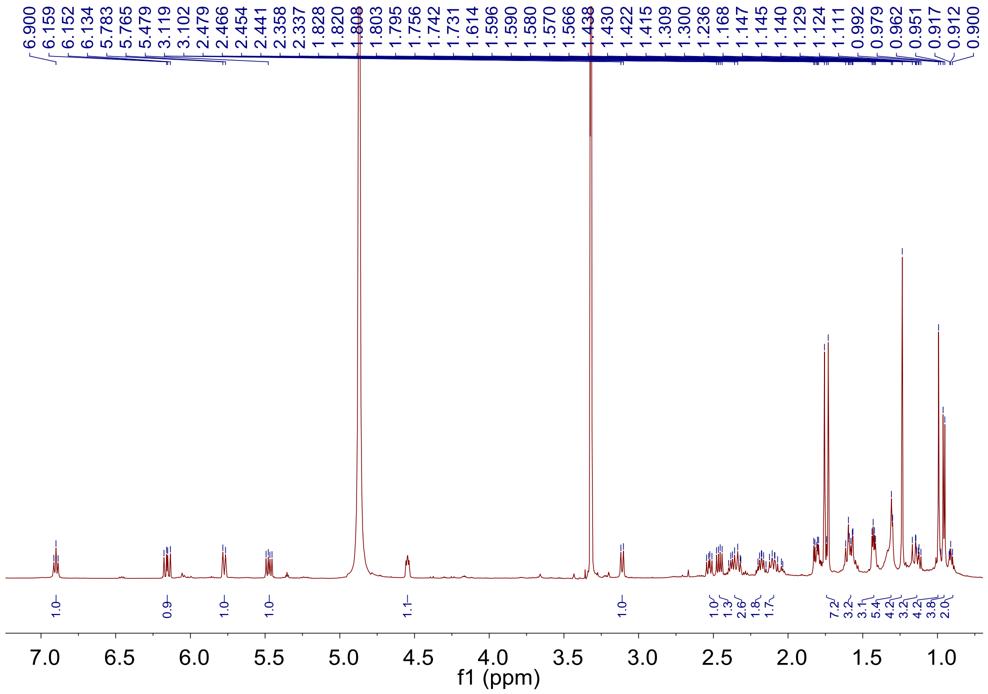

Fig. S44 ^{1}H-NMR spectrum of compound **5** (in CD_3OD)

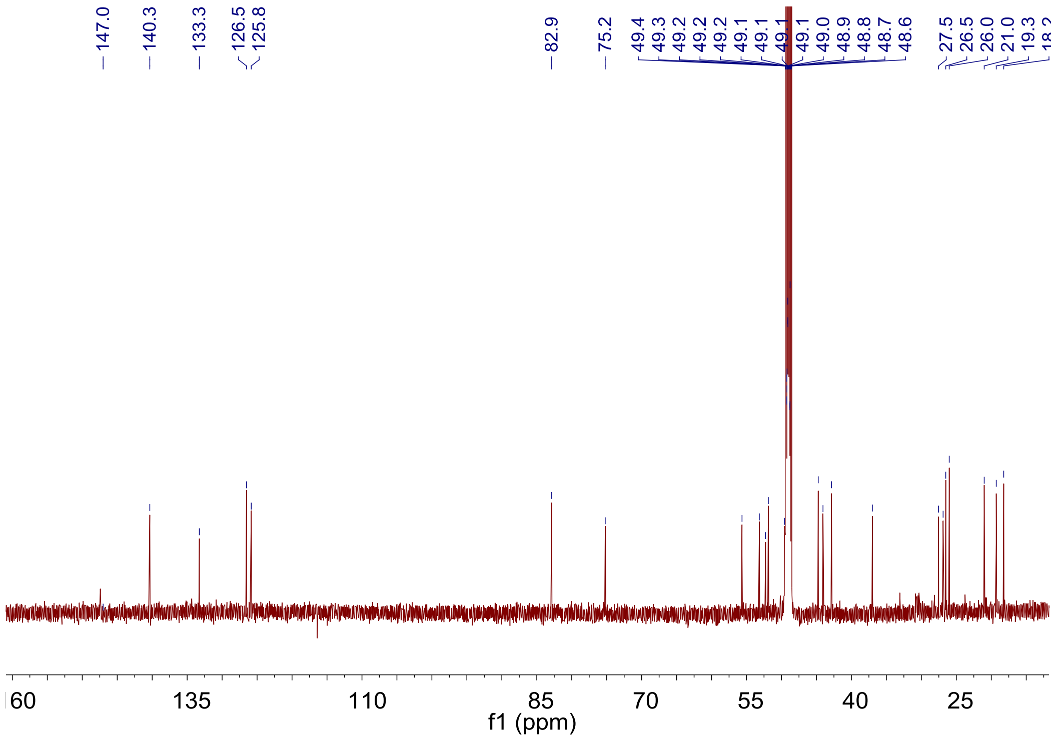

Fig. S45 ^{13}C-NMR spectrum of compound **5** (in CD_3OD)

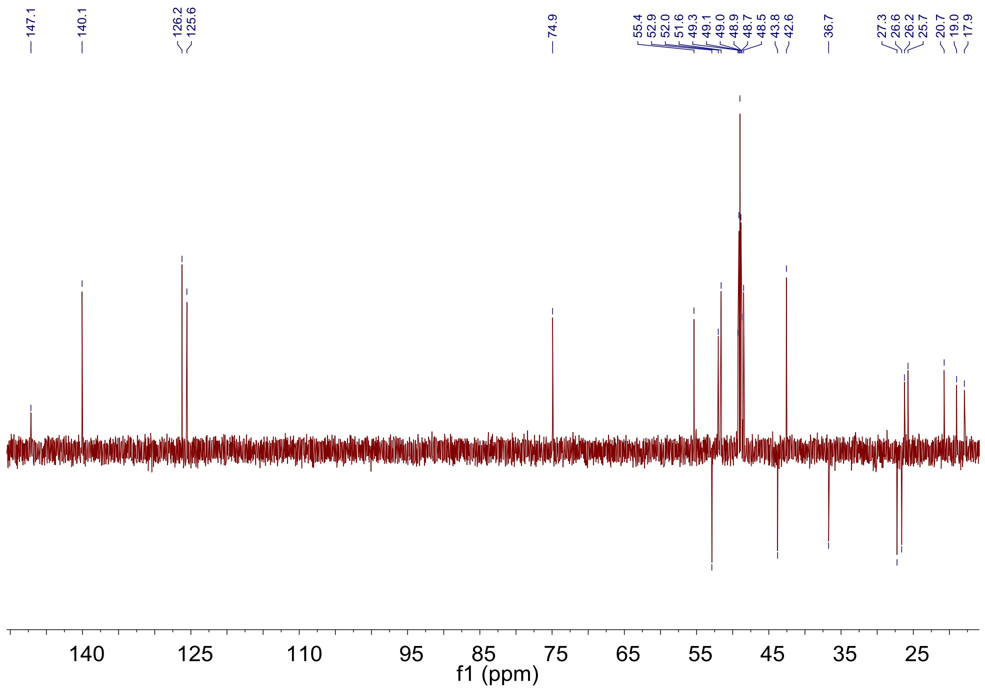

Fig. S46 DEPT135 spectrum of compound **5** (in CD_3OD)

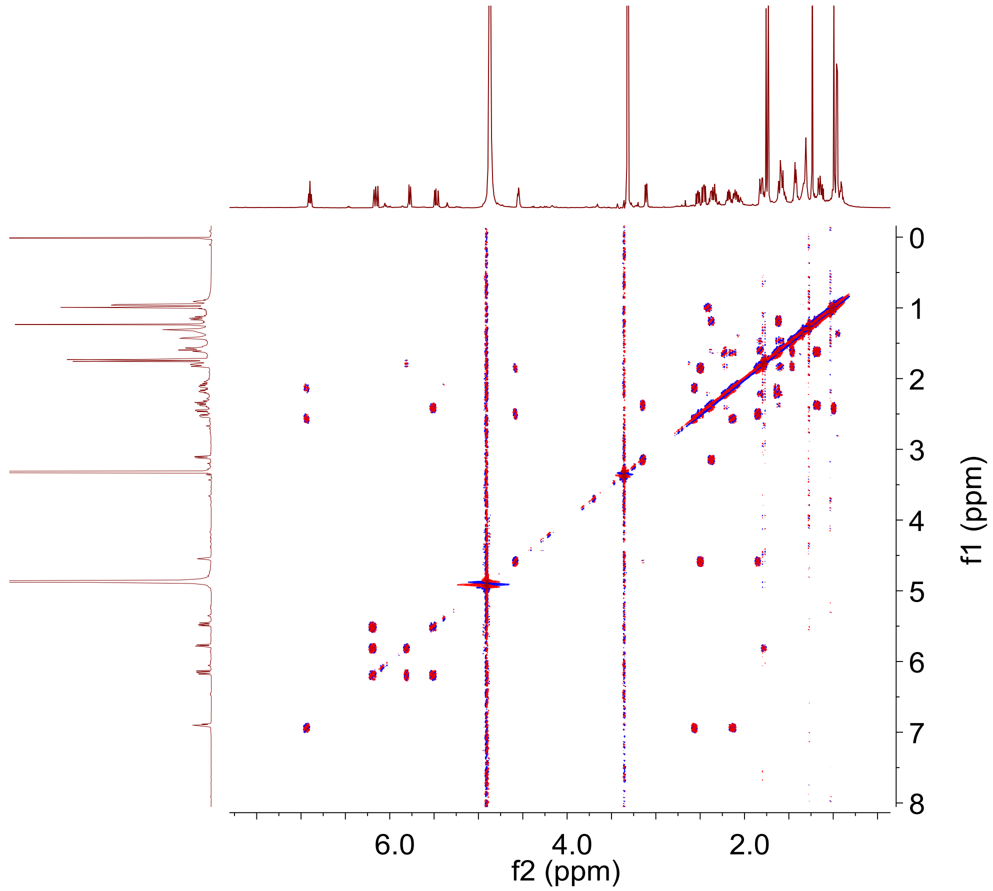

Fig. S47 ^{1}H-^{1}H COSY spectrum of compound **5** (in CD_3OD)

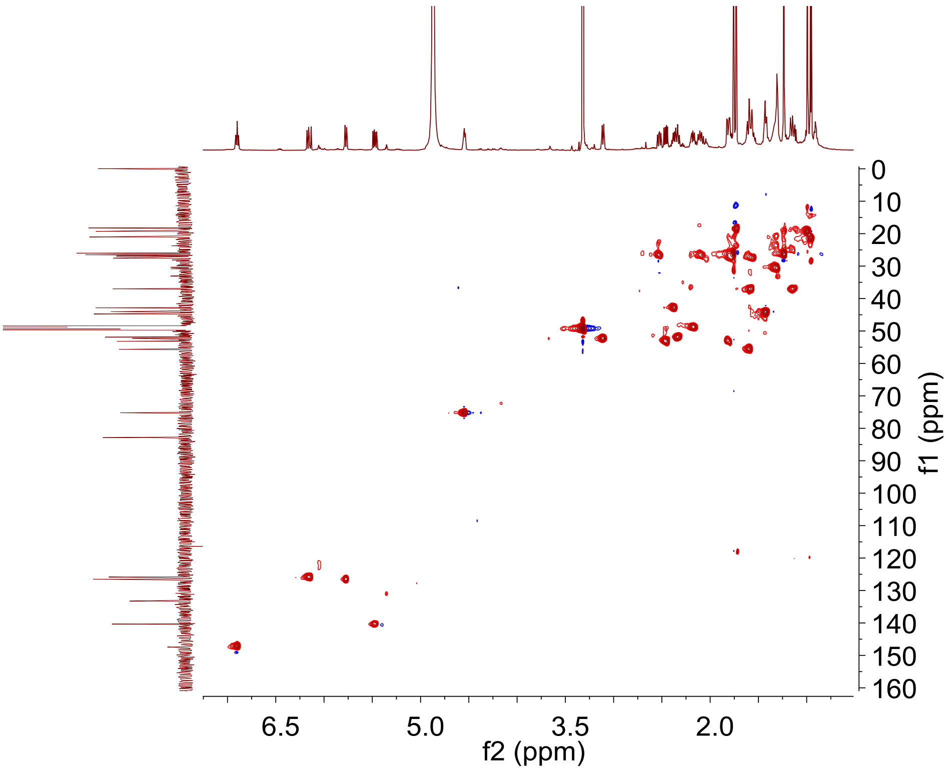

Fig. S48 HSQC spectrum of compound **5** (in CD_3OD).

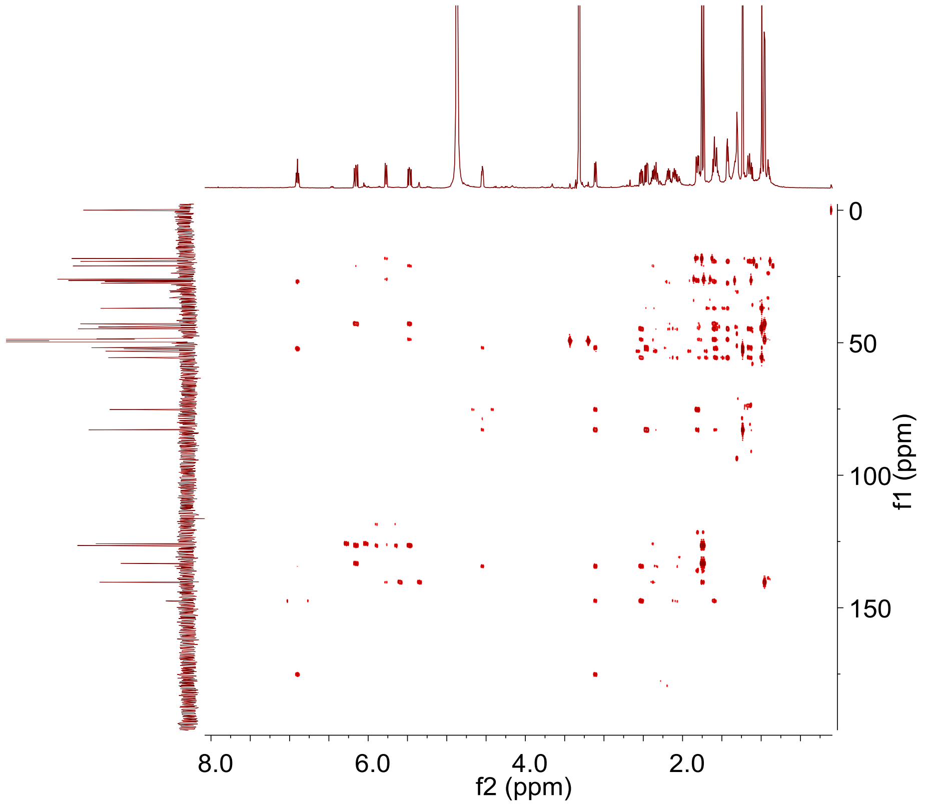

Fig. S49 HMBC spectrum of compound **5** (in CD3OD).

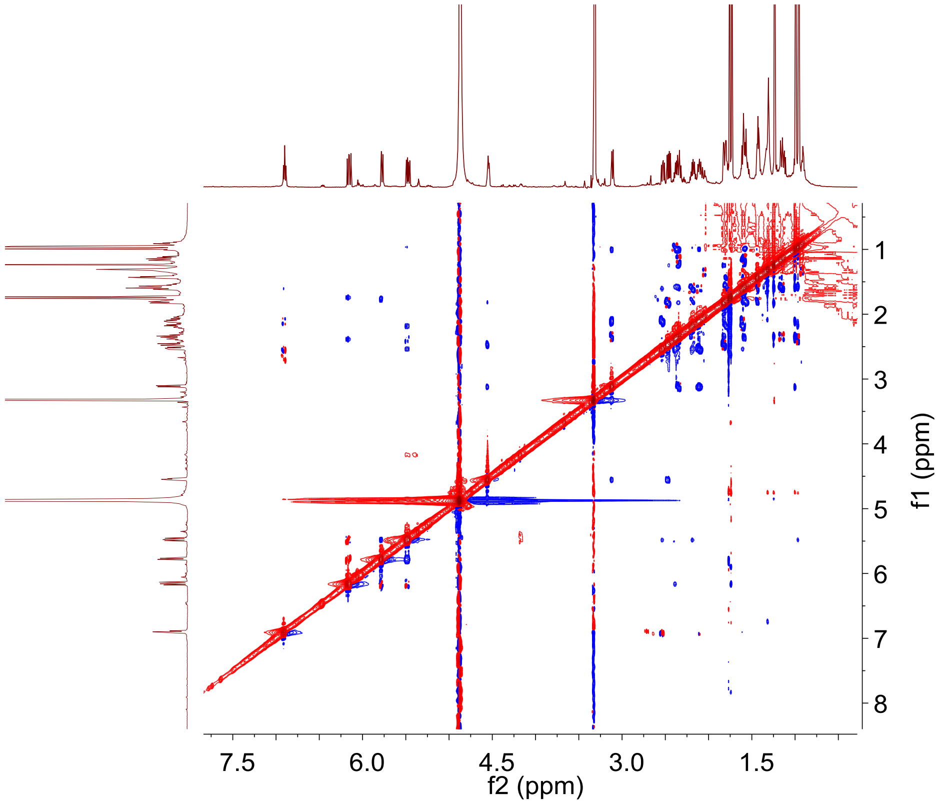

Fig. S50 ROESY spectrum of compound **5** (in CD3OD).

Fig. S51 Cultural and microscopic morphology of strain WHU0154

a. Cultural characteristics (7 days, front) of strain WHUF0154 on a Potato Dextrose Agar medium at 28 °C. The diameter of the colony is 60~65mm. The front of the colony is white, velvety texture, no exudate, and the center of the colony turns light brown-gray when grow old. The reverse side of the colony is gray.

b. The conidiogenous structures of strain WHU0154 (400×). The conidiophore originates from aerial hyphae, with a diameter of 3.8-5 μm, smooth, and the top is slightly enlarged to form a flask-shaped vesicle with a diameter of 7.5-8.8 μm. The upper part to 3/4 of the surface is fertile; the sporulation structure is double-layered, the stem base is 3-4×2-3μm; the bottle stem is 6-7×3-4μm; the conidia are spherical, with a diameter of 2-3μm, smooth.

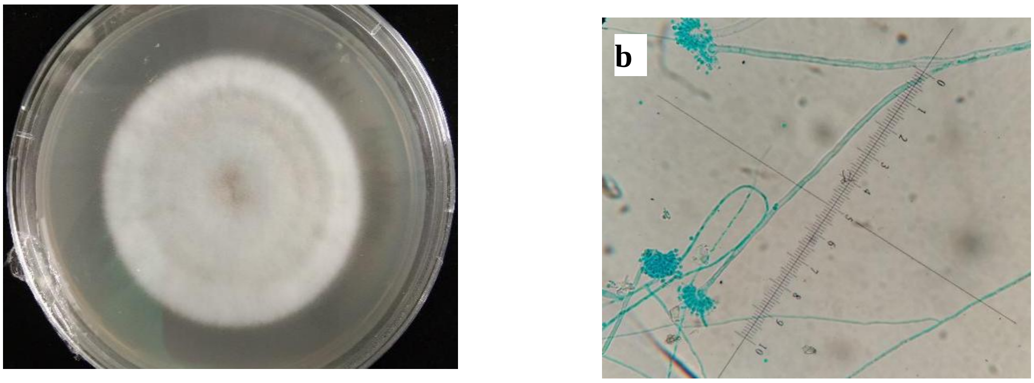

Supplement: Supplementary file 1 [file marinedrugs-18-00575-s001.pdf]
